# Exome-wide association study reveals novel susceptibility genes to sporadic dilated cardiomyopathy

**DOI:** 10.1371/journal.pone.0172995

**Published:** 2017-03-15

**Authors:** Ulrike Esslinger, Sophie Garnier, Agathe Korniat, Carole Proust, Georgios Kararigas, Martina Müller-Nurasyid, Jean-Philippe Empana, Michael P. Morley, Claire Perret, Klaus Stark, Alexander G. Bick, Sanjay K. Prasad, Jennifer Kriebel, Jin Li, Laurence Tiret, Konstantin Strauch, Declan P. O'Regan, Kenneth B. Marguiles, Jonathan G. Seidman, Pierre Boutouyrie, Patrick Lacolley, Xavier Jouven, Christian Hengstenberg, Michel Komajda, Hakon Hakonarson, Richard Isnard, Eloisa Arbustini, Harald Grallert, Stuart A. Cook, Christine E. Seidman, Vera Regitz-Zagrosek, Thomas P. Cappola, Philippe Charron, François Cambien, Eric Villard

**Affiliations:** 1 Sorbonne Universités, UPMC Univ Paris 06, INSERM UMR-S1166, Team Genomics & Pathophysiology of Cardiovascular Diseases, Paris, France; 2 ICAN Institute for Cardiometabolism and Nutrition, Paris, France; 3 Institute of Gender in Medicine and Center for Cardiovascular Research, Charite University Hospital, and DZHK, Berlin, Germany; 4 Institute of Genetic Epidemiology, Helmholtz Zentrum München—German Research Center for Environmental Health, Neuherberg, Germany; 5 Department of Medicine I, Ludwig-Maximilians-University Munich, Munich, Germany; 6 DZHK (German Centre for Cardiovascular Research), Partnersite Munich Heart Alliance, Munich, Germany; 7 INSERM, UMR-S970, Department of Epidemiology, Paris, France; 8 Université Paris Descartes, Sorbonne Paris Cité, Faculté de Médecine, Paris, France; 9 Penn Cardiovascular Institute and Department of Medicine, Perelman School of Medicine, University of Pennsylvania, Philadelphia, PA, United States of America; 10 Department of Genetic Epidemiology, University of Regensburg, Regensburg, Germany; 11 Department of Medecine and Genetics Harvard Medical School, Boston, MA, United States of America; 12 Royal Brompton Hospital, London, United Kingdom; 13 Research Unit of Molecular Epidemiology, Helmholtz Zentrum München—German Research Center for Environmental Health, Neuherberg, Germany; 14 Institute of Epidemiology II, Helmholtz Zentrum München—German Research Center for Environmental Health, Neuherberg, Germany; 15 German Center for Diabetes Research, Neuherberg, Germany; 16 Center for Applied Genomics, Children’s Hospital of Philadelphia, Philadelphia, PA, United States of America; 17 Institute of Medical Informatics, Biometry and Epidemiology, Chair of Genetic Epidemiology, Ludwig-Maximilians-Universität, Munich, Germany; 18 Medical Research Council Clinical Sciences Centre, Faculty of Medicine, Imperial College London, London, United Kingdom; 19 Howard Hughes Medical Institute, Chevy Chase, MD, United States of America; 20 AP-HP, Georges Pompidou European Hospital, Pharmacology Department, Paris, France; 21 INSERM U1116, Université de Lorraine, Nancy, France; 22 AP-HP, Georges Pompidou European Hospital, Cardiology Department, Paris, France; 23 Deutsches Herzzentrum München, Technische Universität München, Munich, Germany; 24 AP-HP, Pitié-Salpêtrière Hospital, Cardiology Department, Paris, France; 25 AP-HP, Hôpital Pitié-Salpêtrière, Centre de Référence des Maladies Cardiaques Héréditaires, Paris, France; 26 IRCCS Fondazione Policlinico San Matteo, Pavia, Italy; 27 National Heart Centre Singapore, Singapore; 28 National Heart and Lung Institute, Imperial College London, London, United Kingdom; 29 Duke-NUS, Singapore; 30 Université de Versailles-Saint Quentin, AP-HP, Hôpital Ambroise Paré, Boulogne-Billancourt, France; Ohio State University Wexner Medical Center, UNITED STATES

## Abstract

**Aims:**

Dilated cardiomyopathy (DCM) is an important cause of heart failure with a strong familial component. We performed an exome-wide array-based association study (EWAS) to assess the contribution of missense variants to sporadic DCM.

**Methods and results:**

116,855 single nucleotide variants (SNVs) were analyzed in 2796 DCM patients and 6877 control subjects from 6 populations of European ancestry. We confirmed two previously identified associations with SNVs in *BAG3* and *ZBTB17* and discovered six novel DCM-associated loci (Q-value<0.01). The lead-SNVs at novel loci are common and located in *TTN*, *SLC39A8*, *MLIP*, *FLNC*, *ALPK3* and *FHOD3*. *In silico* fine mapping identified *HSPB7* as the most likely candidate at the *ZBTB17* locus. Rare variant analysis (MAF<0.01) demonstrated significant association for *TTN* variants only (P = 0.0085). All candidate genes but one (*SLC39A8*) exhibit preferential expression in striated muscle tissues and mutations in *TTN*, *BAG3*, *FLNC* and *FHOD3* are known to cause familial cardiomyopathy. We also investigated a panel of 48 known cardiomyopathy genes. Collectively, rare (n = 228, P = 0.0033) or common (n = 36, P = 0.019) variants with elevated *in silico* severity scores were associated with DCM, indicating that the spectrum of genes contributing to sporadic DCM extends beyond those identified here.

**Conclusion:**

We identified eight loci independently associated with sporadic DCM. The functions of the best candidate genes at these loci suggest that proteostasis regulation might play a role in DCM pathophysiology.

## Introduction

Dilated cardiomyopathy (DCM) is a heart muscle disease characterized by left ventricular dilatation and systolic dysfunction in the absence of abnormal loading conditions or coronary artery disease (CAD). DCM is a major cause of sudden cardiac death and heart failure often requiring heart transplantation, its population-frequency is estimated to be around 1/500 [1]. Genetic studies of familial DCM have identified rare causal variants in more than 50 genes [2]. Genome-Wide Association Studies (GWAS) of sporadic DCM have revealed a few common variants associated with the disease [3]. A locus on chromosome 1 encompassing *ZBTB17*, *HSPB7* and *CLCNKA*, was replicated in several studies [3,4]. However, attempts to correlate the functions of these genes to DCM remained inconclusive[5]. In our prior GWAS, we also identified an association with a missense variant in *BAG3* and demonstrated the implication of *BAG3* mutations in familial DCM [3].

Our prior GWAS had a limited sample size and used a genome-wide tagging array to estimate allele frequencies in stratified pools of DNA. We now report the results of an extended study in 6 populations of European ancestry, using the *Illumina Human Exome Beadchip* which mostly targets variants altering protein sequence (http://genome.sph.umich.edu/wiki/Exome_Chip_Design). We report association analyses for single variants, candidate regions and a panel of 48 genes implicated in familial cardiomyopathy [2].To maximize power and because DCM is a relatively rare disease we conducted exome-wide genotyping in all available patients and controls instead of using a two-step discovery/replication design.

## Methods

See ***S1 Methods*** for details, in brief:

### Ethics statement

Written informed consent was obtained from all study participants. All samples were collected in accordance with the Helsinki declaration and study protocols were approved by the ethics committees of the participating centers: UK population: Southampton and south west Hampshire research ethics committee (09/H0504/104); USA population 1: The Partners Human Research Committee, IRB of Partners Healthcare, Brigham and Women's Hospital; USA population 2: MAGNet study—University of Pennsylvania Institutional Review Board; Eurogene Ethics: CPP comité de protection des personnes dans la recherche biomédicale, Faculty hospital Pitié-Salpêtrière, Paris (ref 66–01); Cardigene Ethics: CPP comité de protection des personnes dans la recherche biomédicale, Faculty hospital Pitié-Salpêtrière, Paris; PHRC Ethics: CPP comité de protection des personnes dans la recherche biomédicale, Faculty hospital Pitié-Salpêtrière, Paris (ref 63–05); German population- Charite University Hospital Ethics Committee, Berlin, Germany.

### Populations included and samples collection

All subject included in the study gave informed consent; the research protocol was approved by local ethic committees and complies with the Declaration of Helsinki. Patients with sporadic idiopathic DCM and controls from six populations of European descent, recruited in Germany, France, UK, USA and Italy, were included in this EWAS. Sporadic DCM was diagnosed according to standard criteria and known secondary causes of the disease as well as familial cases (when relevant information was available) were excluded. Control subjects from the same country of origin were selected for each group of patients except for the MAGNet Study patients (USA2 population) for whom a control group was artificially defined by subsampling 1,000 individuals from the German control group (see ***S1 Methods—Variant-level analysis***).

### Genotyping and data preprocessing

Genotyping was done with *Illumina HumanExome BeadChips* using a standard protocol. Quality control was performed with the 1.9 version of the *PLINK* software [6] and in the *R* version 3.1 environment. Markers with genotyping success rate <99% and samples with <99% of markers available were excluded.

### Protein interaction study

HEK293 cells were transfected with vectors encoding for GFP-tagged BAG3 or GFP (as a negative control). 48h post-transfection cell lysates were subjected to 2 independent protein interaction analyses. For GST pull-down experiments, HEK293 cells were transfected with vectors expressing GFP alone or GFP-tagged BAG3 proteins. Total cell lysates were incubated with Glutathione Sepharose beads complexed with GST-HspB7 recombinant proteins. GST tagged HspB7 proteins were purified from a bacterial expression system (BL21 *E*. *coli*). For co-immunoprecipitation, HEK293 cells were co-transfected with Flag-HSPB7 and GFP or GFP-BAG3 vectors. Total cell lysates were incubated with magnetic Protein A Dynabeads and subjected to immunoprecipitation using an anti-GFP antibody. Input fractions, GST-pull down and GFP-coimmunoprecipitated proteins, were revealed by Western-Blot.

### Statistical analysis

All statistical models were adjusted on age and gender and on the 20 first principal components (PCs) estimated from the genetic relatedness matrix (GRM) [7] to account for a possible population stratification.

### Variant-level analysis

Association between case-control status and each variant was assessed using logistic regression, variant effect being modeled either as additive or dominant in *PLINK*. Homogeneity of effects of DCM-associated variants across populations was tested by a meta-analysis of population-specific data. To account for false discovery rate, a Q-value (*R/Qvalue* package) threshold of 0.01 was chosen. ***S1A Table*** reports the power of our study for various MAF and allelic risk.

### Region and gene set-level analyses

We used *SKAT* [8] to evaluate the global contribution of sets of variants. The analysis was performed for rare (MAF<0.01), common, and all variants combined. Regional analyses were centered on the candidate regions discovered in the variant-level analyses. For the gene-set analysis, genes known to be implicated in familial cardiomyopathy were identified [2] and all available variants on these genes were tested both at the gene level and as a whole. ***S1B Table*** reports the power of our study for the gene set analyzes.

### Fine mapping by imputing variants in the regions of interest

To identify non-genotyped variants in strong linkage disequilibrium (LD) with the lead-SNVs, imputation was performed across regions encompassing the loci identified in the variant-level analysis [9].

### Bioinformatics resources

Variant functionality was assessed using the *Combined Annotation Dependent Depletion* (*CADD*) framework [10]. We also determined tissue-specific gene expression *in silico* using the Genotype-Tissue Expression (*GTEx*) database [11].

## Results

**Fig 1:**
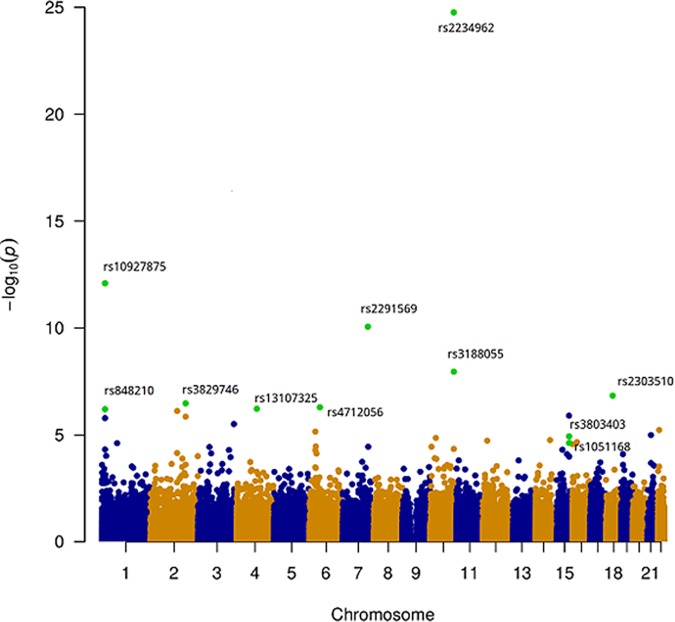
Manhattan Plot of association P-values. 95,499 variants were investigated for association with DCM by logistic regression analysis. Associations are summarized in a Manhattan plot (R/qqman package) which displays the eleven SNVs significantly associated with DCM (Q-values < 0.01) as green dots. Note that the applied logistic model assumed an additive mode of inheritance. For variants on chromosome 15 in the *ALPK3* region, a dominant mode of inheritance was better supported by the data (see Table 1 for corresponding P-values)

**Fig 2:**
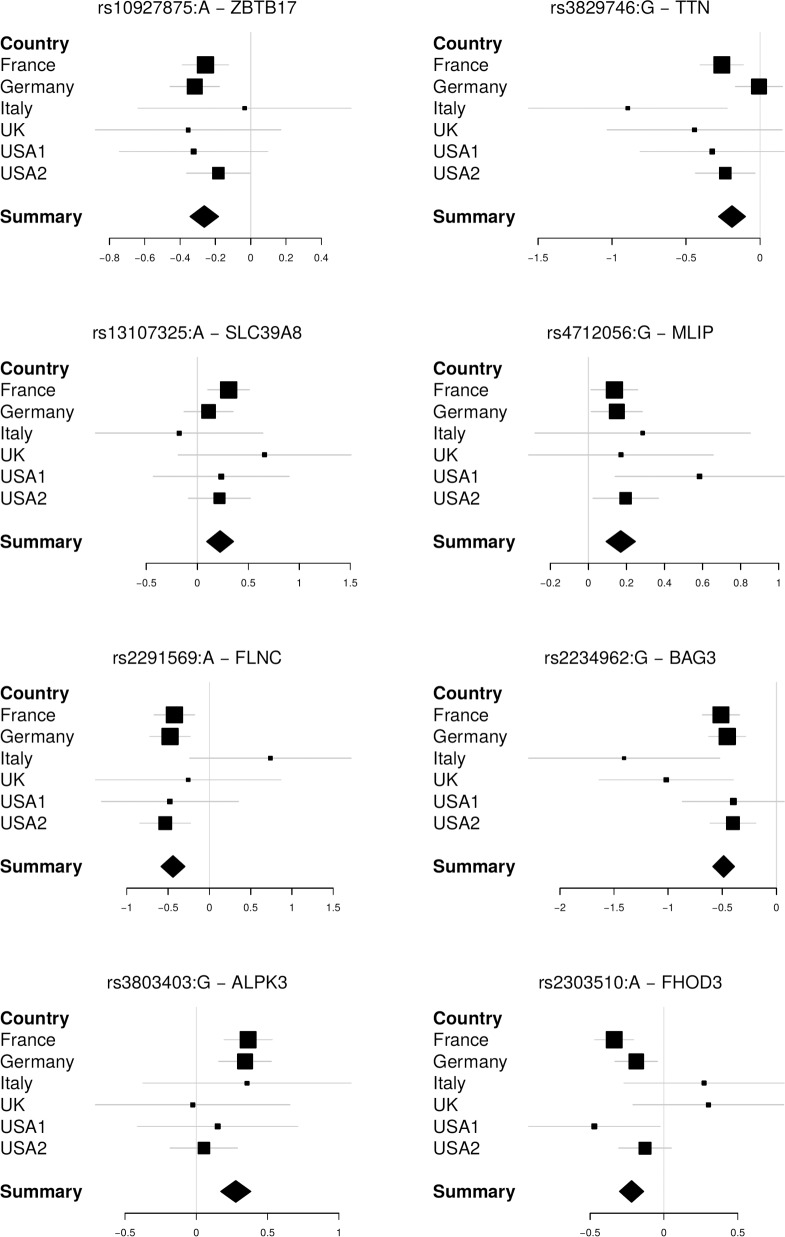
Forest Plots of odd-ratios (log) in the different populations. The results show that the associations were largely homogeneous across populations (See also heterogeneity column in Table 2)

The study included 2796 DCM patients (643 females) and 6877 control subjects (3045 females) (***S2 Table***). After exclusion of variants that were monomorphic in cases and controls, 95499 SNVs were available for analysis. At 1% Q-value, 11 SNVs at 8 distinct loci are significantly associated to DCM (***Fig 1, Table 1***). Re-analysis of the data after removal of the 85 cases with identified familial DCM forms did not modify the results (S7 Table). Two of these loci were previously identified (*ZBTB17-HSPB7* and *BAG3*) and six are novel (*TTN*, *SLC39A8*, *MLIP*, *FLNC*, *NMB-ALPK3*, *FHOD3*). The QQ-plot of association statistics (***S1 Fig***) shows that population stratification was apparently well-controlled (lambda = 0.991). Population-specific Manhattan and QQ-plots are separately reported in ***S2 Fig***. Based on the QQ-plot and lambda value (0.969) for the USA2 cohort, the reconstructed control group for the MAGNet DCM cases appears appropriate. For all loci, the effect of the lead SNP is quite homogeneous across populations (***Fig 2***). Ten of the 11 SNVs encode missense residues, as expected from the enrichment of the exome array for this category of variants.

**Table 1:** Variants associated with DCM.

	MAF	OR (95% CI)	P-value	Q-value	P-Het	Gene	AA Change
rs848210	A: 0.45 (0.39–0.49)	1.182 (1.11–1.26)	6.3x10^-07^	6.0x10^-03^	0.5	SPEN	N/D
rs10927875	A: 0.31 (0.26–0.37)	0.768 (0.71–0.83)	8.1x10^-13^	3.9x10^-08^	0.83	ZBTB17	-
rs3829746	G: 0.23 (0.21–0.24)	0.810 (0.75–0.88)	3.4x10^-07^	4.6x10^-03^	0.039	TTN	I/V
rs13107325	A: 0.08 (0.07–0.12)	1.348 (1.20–1.52)	6.0x10^-07^	6.0x10^-03^	0.61	SLC39A8	A/T
rs4712056	G: 0.35 (0.34–0.36)	1.191 (1.11–1.28)	5.1x10^-07^	6.0x10^-03^	0.53	MLIP	V/I
rs2291569	A: 0.08 (0.05–0.09)	0.651 (0.57–0.74)	8.7x10^-11^	2.8x10^-06^	0.27	FLNC	R/Q
rs2234962	G: 0.19 (0.15–0.22)	0.620 (0.57–0.68)	1.7x10^-25^	1.6x10^-20^	0.14	BAG3	C/R
rs3188055	G: 0.34 (0.33–0.35)	1.223 (1.14–1.31)	1.1x10^-08^	2.6x10^-04^	0.84	INPP5F	N/D
rs1051168	A: 0.30 (0.27–0.35)	1.273 (1.16–1.40)	4.1x10^-07^	4.9x10^-03^	0.32	NMB	P/T
rs3803403	G: 0.30 (0.28–0.35)	1.276 (1.16–1.40)	2.9x10^-07^	4.0x10^-03^	0.29	ALPK3	T/S
rs2303510	A: 0.31 (0.29–0.34)	0.824 (0.77–0.89)	1.5x10^-07^	2.3x10^-03^	0.023	FHOD3	V/I

For rs1051168 (*NMB*) and rs3803403 (*ALPK3*) the result of the dominant model is shown as it is better supported than the additive test which is presented for all other variants. The minor frequency allele is the effect allele.

MAF: Minor allele frequencies (ranges across 6 populations).

OR: Odds-Ratio estimated using a logistic model adjusted on gender and first 20 PCs.

Q-value threshold (0.01)

Het: test of homogeneity of effects across populations.

*SPEN*: spen family transcriptional repressor; *ZBTB17*: zinc finger and BTB domain containing 17; *TTN*: titin; *SLC39A8*: solute carrier family 39 (zinc transporter), member 8; *MLIP*: muscular LMNA-interacting protein; FLNC: filamin C, gamma; *BAG3*: BCL2-associated athanogene 3; *INPP5F*: inositol polyphosphate-5-phosphatase F; *NMB*: neuromedin B; *ALPK3*: alpha-kinase 3; *FHOD3*: formin homology 2 domain containing 3.

### ZBTB17-HSPB7 locus

**Fig 3:**
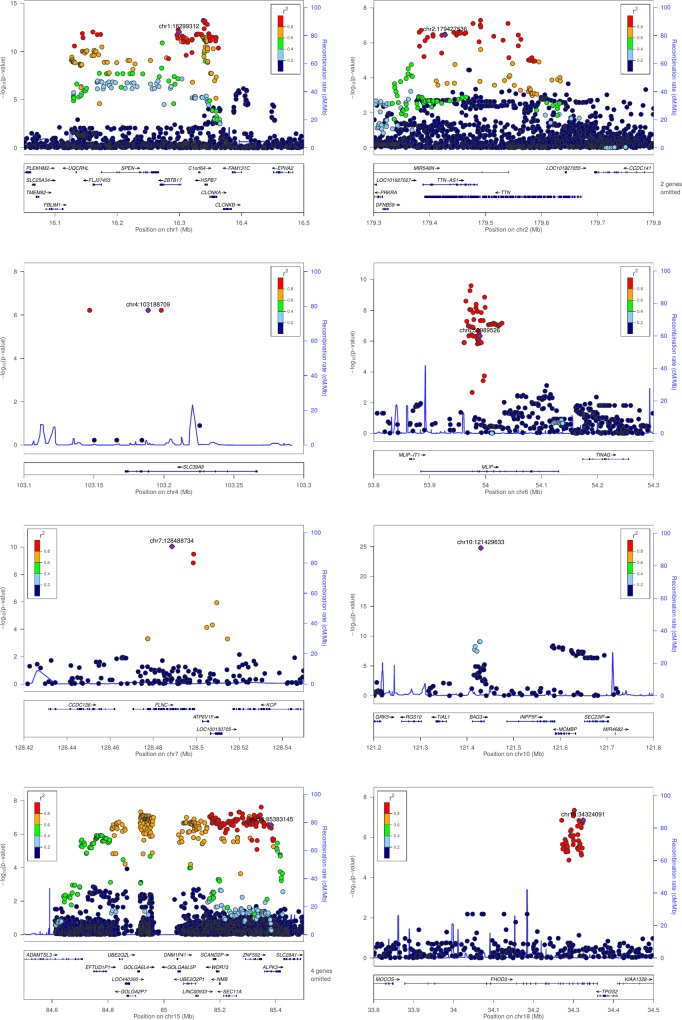
Regional plots of association at 8 loci. P-values, obtained from logistic regression analysis of genotyped and imputed variants in genomic regions demonstrating significant association with DCM are depicted. In each region, the genotyped lead-SNV is identified by its position and the other variants are colored to reflect their LD with the lead-SNV.

**Fig 4:**
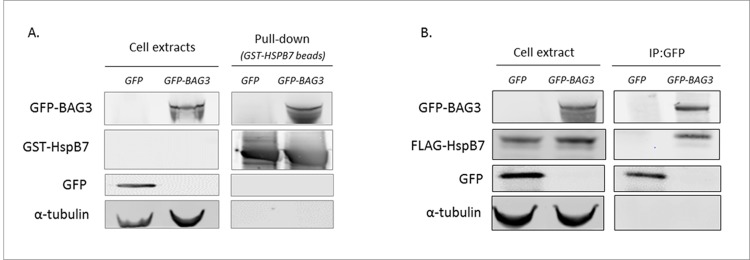
BAG3 interacts with HspB7. (A) GST Pull-Down showing interaction of GFP-BAG3 expressed in HEK293 cells and recombinant GST-HSPB7. GFP-BAG3 was expressed in HEK293 cells (cell extract panel) and and GST-HSPB7 was produced in a bacterial expression system. GST-HSPB7 co-sediment with GFP-BAG3 but not with GFP alone indicating specific BAG3/GST-HSPB7 interaction (Pull-Down panel). (B) Co-immunoprecipitation experiment showing interaction of Flag-HSPB7 and GFP-BAG3 in HEK293 cells. GFP alone or GFP-BAG3 were co-expressed together with Flag-HSPB7 in HEK293 cells (cell extract panel) and subjected to immunoprecipitation with an antibody against GFP. Only GFP-BAG3 immunoprecipitated with FLAG-HSPB7 (IP:GFP panel). Western blottings in (A) and (B) used HSPB7 (for GST-HSPB7), GFP (for GFP and GFP-BAG3), and α-tubulin specific antibodies.

The lead SNV, *rs10927875* (ZBTB17, c.-3+222G>A, MAF = 0.31), is located in an intron of *ZBTB17*. As observed in our earlier GWAS [3], this SNP confers a reduced risk of DCM (OR = 0.77 (0.71–0.83), P = 8.1x10^-13^). Imputation revealed a large number of SNVs in strong LD with *rs10927875* (***S3A Table***), but as a result of the "Yin-Yang" haplotypic structure of the region we could not determine the most likely causal variant or gene (***Fig 3A***). However, several imputed SNVs located within or downstream the heat shock 27kDa protein family, member 7 gene (*HSPB7*) had slightly more significant P-values than *rs10927875* (***S3A Table***). Generally, SNVs within *HSBP7* had more deleterious *CADD* scores than those within *ZBTB17* or *CLCNKA* (another candidate within the region [4]). In addition, *GTEx* analysis showed that *HSPB7* is mostly expressed in the heart and skeletal muscles. Using GST pull-down experiment and co-immunoprecipitation we also observed that recombinant BAG3 interacts with HSPB7 (***Fig 4***). Overall, this suggests that *HSPB7* is the best candidate to explain the association of variants in this region with DCM.

### TTN locus

Titin is a major component of the sarcomere and an important familial DCM gene [12]. The lead SNV, *rs3829746* in (*TTN*, c.56704A>G, p.Ile18902Val, MAF = 0.23) confers a reduced risk of DCM (OR = 0.81 (0.75–0.88), P = 3.4x10^-7^). A regional plot of all variants (genotyped and imputed) shows that several are in tight LD with the lead SNV (***Fig 3B)*** and associated with DCM. Among these SNVs, *rs2042996* (p.Thr21403Ile) has the highest *CADD* score (20.1) (***S3B Table***). According to *GTEx rs3829746* is associated with reduced *TTN* expression in the left ventricle (P = 0.04) and atrial appendage (P = 0.006). Unlike *TTN* truncating variants that cause DCM, the DCM-associated TTN missense variants present on the exome-array are independent of *TTN* exon usage. (***S1 Methods—Exon usage in TTN***).

### SLC39A8 locus

Solute carrier family 39 member 8 gene encodes encodes a transmembrane metal-ion transporter exhibiting highly pleiotropic effects. The lead SNV *rs13107325* (*SLC39A8*, c.1171G>A, p.Ala391Thr, MAF = 0.08) confers an increased risk of DCM (OR = 1.35 (1.20–1.52), P = 6.0x10^-7^). Imputation reveals little genetic variability at this locus (***Fig 3C, S3C Table***) and *rs13107325* has the highest *CADD* score (35.0), suggesting that it might be the causal variant. The possible implication of this locus in DCM is intriguing, given that *SLC39A8* is minimally expressed in the heart.

### MLIP locus

Muscular Lamin A (LMNA)-interacting protein interacts with LMNA, a structural component of nuclear lamina known to be implicated in familial DCM [13]. The lead SNV *rs4712056* (*MLIP*, c.475G>A, p.Val159Ile, MAF = 0.35) is associated with an increased risk of DCM (OR = 1.19 (1.11–1.28), P = 5.1x10^-7^). Several imputed variants in the gene are more strongly associated with DCM than *rs4712056* (***Fig 3D, S3D Table***). All of them are intronic or located upstream of the sequence encoding the short cardiac transcript of *MLIP*. The strongest association implicates *rs35182047*, a small intronic insertion (c.64-12401_64-12400insAT). Although the *CADD* score of this variant is modest (2.33), its DCM-associated risk (OR = 1.30 (1.20–1.40, P = 2.5x10^-10^) is substantially higher than that of *rs4712056*.

### FLNC locus

Filamin C is an actin-crosslinking protein, specifically expressed in cardiac and skeletal muscles. The lead SNV *rs2291569* (*FLNC*, c.4700G>A, p.Arg1567Gln, MAF = 0.09) is associated with a reduced risk of DCM (OR = 0.65 (0.57–0.74), P = 8.7x10^-11^). Fine mapping analysis shows that two imputed variants located in the 3'UTR of *FLNC* are in strong LD with *rs2291569* and exhibit similar ORs (***Fig 3E, S3E Table***).

### BAG3 locus

This locus was the second one identified in our earlier GWAS.(3) *rs2234962* (*BAG3*,c.451T>C, p.Cys151Arg, MAF = 0.19) confers a reduced risk of DCM (OR = 0.62 (0.57–0.68), P = 1.7x10^-25^). A nearby SNV, *rs3188055*, located in the *INPP5F* is no longer significant after conditioning on *rs2234962*. When also considering imputed variants at the locus, *rs2234962* is by far the most significant and it also has the highest *CADD* score (24.2) (***Fig 3F, S3F Table***).

### NMB-ALPK3 locus

Two missense variants in strong LD (r^2^ = 0.82), *rs1051168* in Neuromedin B (*NMB*, c.217C>A, p.Pro73Thr, MAF = 0.30) and *rs3803403* in alpha-kinase 3 (*ALPK3*, c.1241C>G, p.Thr414Ser, MAF = 0.30) are associated with DCM at this locus. The minor allele at both loci exerts a dominant effect on DCM risk (OR = 1.27 [1.16–1.40]). Fine-mapping analysis identifies a single major haplotypic structure encompassing *NMB*, *ALPK3* and several other genes (***Fig 3G***). *CADD* scores do not orient towards causal variants among these tightly associated SNVs (***S3G Table***). However, *GTEx* indicates that the genes in the interval are not or very lowly expressed in the heart or skeletal muscle, except *ALPK3* which is almost exclusively expressed in these tissues. *ALPK3* encodes a nuclear kinase implicated in the differentiation of cardiomyocyte and *Alpk3*-deficient mice develop cardiomyopathy [14].

### FHOD3 locus

Formin homology 2 domain containing 3 regulates actin assembly and sarcomere organization in striated muscles. The lead SNV *rs2303510* (*FHOD3*, NM_025135.4:c.3591G>A, NP_079411.2:p.Val1151>Ile, MAF = 0.31) is associated with a reduced risk of DCM (OR = 0.82 (0.77–0.89), P = 1.5x10^-07^). Imputation identifies several SNVs, most of them intronic, in strong LD with *rs2303510* and clustered within a relatively narrow region of the *FHOD3* sequence (***Fig 3H, S3H Table***). Among these SNVs, the highest *CADD* score (22.8) is observed for the missense *rs2303510* variant.

### Tissue expression of identified candidate genes

According to *GTEx*, with the exception of *SLC39A8*, the best candidate genes at the DCM-associated loci are all preferentially expressed in heart or skeletal muscle tissues. However, except for *TTN*, their expression levels in these tissues are unaltered by the lead-SNVs.

### Gene-level analysis

Because analysis of single SNVs lacks power for detecting association with rare variants, we tested whether genotyped variants in the candidate regions were collectively associated with DCM. A first analysis showed a significant association of rare variants (MAF<0.01) at the *ZBTB17* and *TTN* loci with DCM (***Table 2***). However, when conditioning on the lead SNP at each locus, only the association of *TTN* rare variants remained significant (P = 0.013). When including all variants (rare and common) in the analysis and conditioning on the lead SNVs, there was still a significant association (P<0.05) at the *ZBTB17*, *TTN* and *BAG3* loci which implies residual associations independent of the lead-SNVs (***Table 2***).

**Table 2:** Gene-level association analysis of genes identified in the SNP-level analysis

Region	number of variant (rare f<0.01)	All variants P-value (adjusted^$^)	Rare variants P-value (adjusted^$^)	Common variants P-value (adjusted^$^)
ZBTB17	54 (47)	1.1x10^-12^ (0.007)	0.046 (0.4)	2.14x10^-12^ (0.0027)
TTN	457 (369)	3.1x10^-06^ (0.0083)	0.0085 (0.013)	1.20x10^-05^ (0.048)
SLC39A8	4 (2)	9.5x10^-05^ (0.23)	0.13 (0.12)	1.65x10^-05^ (0.5)
MLIP	24 (15)	1.2x10^-04^ (0.19)	0.91 (0.81)	1.68x10^-06^ (0.049)
FLNC	72 (61)	2.3x10^-04^ (0.46)	0.32 (0.4)	2.34x10^-05^ (0.48)
BAG3	32 (22)	2.6x10^-20^ (0.037)	0.23 (0.53)	4.36x10^-23^ (0.0094)
ALPK3	42 (33)	2.5x10^-02^ (0.8)	0.55 (0.53)	3.71x10^-03^ (0.89)
FHOD3	40 (30)	6.9x10^-04^ (0.27)	0.083 (0.086)	7.80x10^-04^ (0.77)

The gene-level analysis was performed using the *R/SKAT_CommonRare* function (See ***S1 Methods***).

^$^ P-values adjusted on lead-SNV.

### Familial DCM gene-set analysis

Sixty genes are reported to be implicated in familial cardiomyopathies [2]. To investigate whether these genes might also have a role in sporadic DCM, we tested their association at the gene level. After discarding *BAG3* and *TTN* and genes harboring no variant in our data set, 48 genes and 608 variants (478 of which are rare) were tested (***S4 Table***). No association was observed for rare variants after Bonferroni correction for 48 genes. For common variants, the only significant association was observed for *MYBPC3* (4 variants, P = 5.7x10^-5^, P<0.003 after Bonferroni correction). In the *MYBPC3* gene, the most significant SNV had a P-value of 3.06x10^-03^. The entire set of 608 variants was associated with DCM (P = 0.0067) and the association was strengthened for variants with a *CADD* severity score > 20 (n = 264, P = 0.0005); both rare (P = 0.0033) and common variants (P = 0.019) contributed to this association (***Table 3***).

**Table 3:** DCM gene set association analysis

variants subset	Number of variants (rare f<0.01)	All variants P-value	Rare variants P-value	Common variants P-value
All	608 (478)	0.0067	0.013	0.091
CADD20	264 (228)	0.0005	0.0033	0.019
CADD25	71 (63)	0.016	0.047	0.064
CADD30	30 (26)	0.064	0.19	0.07

All variants on 48 familial DCM genes (excluding *BAG3* and *TTN*) also categorized by *CADD* severity scores.

## Discussion

In this EWAS of sporadic DCM we confirmed associations with variants in *ZBTB17-HSPB7* and *BAG3* and identified six novel loci. Statistical analyses, cardiac tissue expression, and physiology suggest that the most likely causal genes are *HSPB7*, *BAG3*, *TTN*, *SLC39A8*, *MLIP*, *FLNC*, *ALPK3* and *FHOD3*.

Our data provide evidence that non-coding variants close to or within *HSPB7* are more likely to account for the observed association at the *ZBTB17* locus. The genetic mechanism linking the risk haplotype to HSPB7 functional modulation in absence of detectable eQTL is unknown. HSPB7 (commonly referred as cardiovascular Heat Shock Protein; cvHSP) is a member of the small HSPB family of molecular chaperones. It is a potent polyglutamin aggregation suppressor that assists the loading of misfolded proteins or small protein aggregates into autophagosomes [15]. In addition, our *in vitro* experiments demonstrate a physical interaction of BAG3 with HSPB7 (***Fig 4***) suggesting functional relationships between the 2 proteins that may be relevant for their genetic implication in DCM pathophysiology. The strongest association with sporadic DCM in our EWAS involved *rs2234962* which encodes a p.Cys151Arg substitution in BAG3. The interaction signal of BAG3 Arg151 and Cys151 isoforms with HSPB7 was similar (data not shown), suggesting no direct effect of the polymorphism on HSPB7 binding The p.Cys151Arg variant is located between two conserved Ile–Pro–Val (IPV) motifs involved in BAG3 complex formation with HSPB6 and HSPB8. Interestingly, a p.Pro209Leu mutation responsible for myofibrillar myopathy associated with cardiomyopathy is located in one of the two IPV motifs [16]. Whether p.Cys151Arg modifies the interaction of BAG3 with HSPBs partners and affects the functional potential of the complex is currently unknown.

Common haplotype-tagging variants of the titin gene were associated with small differences in DCM risk in this EWAS. This extends the spectrum of *TTN* genetic variants that affect DCM risk, from highly penetrant mutations responsible for familial DCM [12] to common haplotypes with low penetrance associated with sporadic DCM. A potential consequence of common DCM-associated *TTN* variants, in line with the pathogenic mechanisms suggested by the candidate genes identified in this EWAS, is the proteotoxic effect of accumulating truncated or aggregate prone mutant TTN in cardiomyocytes.

The *rs13107325* SNV in *SLC39A8* has been shown in GWAS to be associated with several traits affecting cardiovascular risk, including blood pressure. It is therefore conceivable that it has systemic consequences that raise the risk of DCM but were not considered in our disease exclusion criteria. Because *SLC39A8* encodes a zinc transporter [17], its association with DCM may also be related to the cardioprotective role of zinc [18].

In the nuclear envelope, MLIP (also known as CIP) directly interacts with the N-terminal region of lamin (LMNA) [19]. Dominant mutations in LMNA cause DCM and other hereditary multisystemic diseases and several pathogenic mutations of LMNA are located in its MLIP interacting domain [20]. In mice, Mlip interacts with Isl1, a transcription factor required for cardiomyocyte differentiation, and represses its transcriptional activity [21]. Notably, the DCM-associated SNV (*rs4712056*, p.Val159Ile) is located within the Isl1-interacting region of MLIP. MLIP has recently been shown to be a key regulator of cardiomyopathy that has potential as a therapeutic target to attenuate heart failure progression [22].

Filamin C is involved in the organization of actin filaments, it serves as a scaffold for signaling proteins and interacts with several Z-disk proteins. *FLNC* mutations in humans and mice cause hypertrophic cardiomyopathy [23] and myofibrillar myopathy, a form of muscular dystrophy with concurrent cardiomyopathy [24]. These pathologies are characterized by myofibrillar disorganization, accumulation of myofibrillar degradation products and ectopic expression of multiple proteins [25]. *FLNC* mutations induce massive protein aggregates within skeletal muscle fibers and altered expression of chaperone proteins and components of proteasomal and autophagic degradation pathways. Interestingly, functional interaction between FLNC and HSPB7 or BAG3, two genes confirmed by this study, have been previously reported [26,27]. In addition to the fact that *BAG3* mutations also causes myofibrillar myopathy it suggests the hypothesis that dysregulation of proteostasis could be a common mechanism underlying myofibrillar myopathy and DCM.

Cardiac FHOD3 plays a crucial role in the sarcomere organization of cardiomyocytes, is essential for heart myofibrillogenesis [28] and is required for the maintenance of the contractile structures in heart muscle. A cardiac isoform of FHOD3 is targeted to thin actin filaments via phosphorylation of tyrosine residue preventing autophagy dependent degradation [29]. Most DCM-associated SNVs in our EWAS are clustered in a region in 3' of *FHOD3* that encodes the Formin FH2 domain of the protein, which is implicated in actin polymerization [30]. A FHOD3 variant, Y1249N, has been reported in a Japanese patient with a dominant form of DCM. *In vivo* functional analysis showed that this variant may impair actin filament assembly, thus providing some support for the implication of *FHOD3* in the pathogenesis of DCM [31].

Alpha-kinase 3 (*ALPK3/MIDORI*) was initially described as a myocyte-specific gene that promotes differentiation of P19CL6 cells into cardiomyocytes [32]. The pattern of expression of *ALPK3* in differentiating cardiomyocytes nucleus is similar to that of transcription factors specific of the cardiogenic lineage [33] but its function is still largely unknown. Recently recessive mutations in *ALPK3* have been reported to cause pediatric DCM [34].

In addition to *TTN* and *BAG3*, *MYBPC3* was present in the cardiomyopathy gene-set [2] and harbored variants associated with sporadic DCM in our EWAS. MYBPC3 is an actin, myosin and titin interacting protein of the M-band of the sarcomere. Mutations in this gene are a major cause of hypertrophic cardiomyopathy and have also been reported in familial forms of DCM [35]. Coding variants in *MYBPC3* may affect actin-myosin interaction [36] and concurrently interfere with the ubiquitin proteasome system and autophagy in humans and animal models [37]. Both mechanisms could account for the association of common SNVs in *MYBPC3* with sporadic DCM.

Considered as a whole, both rare and common variants with elevated *CADD* scores in the cardiomyopathy gene-set were associated with sporadic DCM (***S3 Table***) indicating that other loci than those found in this EWAS are involved in sporadic DCM, however identifying the responsible genes will require larger studies.

### Proteostasis might be important for DCM

Three of the DCM-associated genes, *FLNC*, *TTN* (through its kinase activity) and cardiac specific *FHOD3* encode maintenance partners of sarcomere and sarcomere-related structures, including Z-disk or F-actin myofibrils [29,38,39], which are disorganized or degraded in experimental models of cardiomyopathy [40]. Moreover the cellular level of FLNC, FHOD3 and TTN kinase targets such as MuRF2, appears regulated by proteostasis mechanisms [26,29,39]. One of these mechanisms, BAG3-associated chaperone-assisted selective autophagy (CASA) is described as a central adaptation mechanism that responds to acute physical exercise and to repeated mechanical stimulation [41]. *BAG3* inactivation also leads to Z-disk disruption in mice and fruit fly [26]. Based on the functional similarities (also pertaining to *HSPB7* and *MYBPC3*) characterizing several of the DCM-associated genes identified in this study, we hypothesize that abnormal cardiomyocyte sarcomere maintenance and regulation of autophagy is a potential mechanism involved in DCM pathophysiology is. Further experimental exploration of this hypothesis may yield novel therapeutic targets for DCM.

### Limitations of this study

This study has some limitations. The recruitment was focused on *a priori* homogeneous sets of patients and controls of European ancestry and outliers were excluded based on genomic data. We also conducted a meta-analysis which did not reveal any significant heterogeneity across populations. Despite these precautions, we cannot fully exclude undetected population stratification. In addition, given the rather low prevalence of DCM, we conducted exome-wide genotyping in all available patients and controls instead of using a two-step discovery/replication design. As a consequence, even if we provide a series of arguments supporting the identified genes as plausible candidates, independent studies would certainly further refine and extend our results. It is likely that a genome-wide tagging array not limited to exon regions would identify other DCM-associated variants and loci than those reported here. Finally, as our power analyses show, our EWAS had limited power to detect the collective effect of rare variants present in our data set at the gene level.

### Conclusion

We identified 6 novel loci associated with sporadic DCM and confirmed two previously reported associations with variants located within the *ZBTB17-HSPB7* and *BAG3* genes. Fine mapping revealed that at the *ZBTB17* locus *HSPB7* is likely the implicated gene. The lead-SNVs at all associated loci are common variants and conditioning on them reduced considerably the associations of other variants in the regions of interest with DCM. We provide evidence that 7 of the DCM-associated genes are very plausible candidates from a pathogenic perspective.

## Supporting information

S1 Methods (DOCX)

S1 Fig QQ Plot of association p-values. (PDF)

S2 Fig Manhattan and QQ plots by population. (PDF)

S3 Fig Endogenous BAG3 interacts with GST-tagged HSPB7 in Hela cells. (TIF)

S1 Table (DOCX)

S2 Table Number of DCM patients and controls. (DOCX)

S3 Table In Silico fine mapping at loci. (DOCX)

S4 Table SKAT analysis of variants in genes known to be associated with familial cardiomyopathy. (DOCX)

S5 Table Summary statistics-additive model. (TXT)

S6 Table Summary statistics-dominant model. (TXT)

S7 Table Association statistics comparison with and without the 90 FDCM cases. (DOCX)
