# Correction: Exome-wide association study reveals novel susceptibility genes to sporadic dilated cardiomyopathy

**DOI:** 10.1371/journal.pone.0229472

**Published:** 2020-02-14

**Authors:** Ulrike Esslinger, Sophie Garnier, Agathe Korniat, Carole Proust, Georgios Kararigas, Martina Müller-Nurasyid, Jean-Philippe Empana, Michael P. Morley, Claire Perret, Klaus Stark, Alexander G. Bick, Sanjay K. Prasad, Jennifer Kriebel, Jin Li, Laurence Tiret, Konstantin Strauch, Declan P. O’Regan, Kenneth B. Marguiles, Jonathan G. Seidman, Pierre Boutouyrie, Patrick Lacolley, Xavier Jouven, Christian Hengstenberg, Michel Komajda, Hakon Hakonarson, Richard Isnard, Eloisa Arbustini, Harald Grallert, Stuart A. Cook, Christine E. Seidman, Vera Regitz-Zagrosek, Thomas P. Cappola, Philippe Charron, François Cambien, Eric Villard

Based on experiments conducted since this article’s [1] publication, the authors have concluded that the protein-protein interaction results reported in Fig 4 are not reliable.

In experiments done after the article was published, the authors observed signals for the negative control indicating that there was unspecific binding to the sepharose beads (S1 File). Furthermore, efforts to replicate the experiment using the transfection/co-immunoprecipitation protocol detailed in the article did not confirm the published results: a specific protein-protein interaction was not observed in co-immunoprecipitation experiments using native neonate cardiomyocyte extracts (S2 File).

Given these findings, the result and conclusion statements referring to a physical interaction between BAG3 and HSPB7 are not supported. Specifically:

- The penultimate sentence of the ZBTB17-HSPB7 locus subsection of the Results: “Using GST pull-down experiment and co-immunoprecipitation we also observed that recombinant BAG3 interacts with HSPB7 (Fig 4).”
- The fifth sentence of the second paragraph of the Discussion section: “In addition, our *in vitro* experiments demonstrate a physical interaction of BAG3 with HSPB7 (Fig 4) suggesting functional relationships between the 2 proteins that may be relevant for their genetic implication in DCM pathophysiology.”
- The seventh sentence of the second paragraph of the Discussion section: “The interaction signal of BAG3 Arg151 and Cys151 isoforms with HSPB7 was similar (data not shown), suggesting no direct effect of the polymorphism on HSPB7 binding.”

A member of *PLOS ONE*’s Editorial Board confirmed that the main conclusions of the article stand are not substantively affected by this issue.

The authors apologize for the errors in the published article.

## Supporting information

S1 File Results of co-immunoprecipitation experiment conducted in HEK cells, showing non-specific binding cells over-expressing BAG and HSPB7. (PPTX)

S2 File Experimental results of co-immunoprecipitations in native rat neonate cardiomyocytes. (PPTX)
