# Supplementary material for: Exome-wide association study reveals novel susceptibility genes to sporadic dilated cardiomyopathy
Source: PLoS One. 2017 Mar 15;12(3):e0172995. doi: 10.1371/journal.pone.0172995 (PMC5351854; doi:10.1371/journal.pone.0172995)
Supplement: S1 Methods — (DOCX) [file pone.0172995.s001.docx]

## Supplementary Methods: "Exome-wide association study reveals novel susceptibility genes to sporadic dilated cardiomyopathy".

### Diagnosis of DCM

Sporadic DCM was diagnosed according to standard criteria.([1](#ref-elliott_classification_2008)–[3](#ref-mestroni_guidelines_1999)) DCM was diagnosed by a low ejection fraction (echocardiography:<45%; MRI: 2 SDs below the age- and sex-adjusted average) and enlarged left ventricle end-diastolic volume/diameter (w>117% of value predicted from age and body surface area on echocardiography, or 2 SDs from the age- and sex-adjusted mean by MRI(2,11,12) in the absence of significant coronary artery disease or intrinsic valvular disease, documented myocarditis, systemic disease, sustained arterial hypertension, or congenital malformation. When appropriate information was available at the initiation of the study, DCM cases were excluded when family history identified at least one affected relative. However, 85 cases were identified in the final panel of cases used for the association analysis.

### Studies description

**CARDIGENE study.** Cases ([4](#ref-charron_identification_1999)) were French patients with a diagnosis of idiopathic DCM (enlarged left ventricle end-diastolic volume/diameter >140 ml/m2 on ventriculography or >34 mm/m2 on echocardiography and low ejection fraction (≤40%) confirmed over a six-month period, in the absence of causal factors such as coronary artery disease (coronary angiography was mandatory if DCM occurred after 35 years of age) or sustained arterial hypertension, intrinsic valvular disease, documented myocarditis, congenital malformation, insulin-dependent diabetes. Only apparently sporadic DCM cases without additional (first degree) relative with DCM were included. All were of European origin (born in France, from parents born in France or neighboring countries). Recruitment was performed in ten hospitals from six French regions (Lille, Lyon, Nancy, Nantes, Paris-Ile de France, and Strasbourg) from September 1994 to February 1996. A total of 423 DCM cases were included (337 men and 86 women, among them 224 had undergone a cardiac transplantation). Mean age of patients at diagnosis was 39.9 ± 6.2 years, mean left ventricular ejection fraction (LVEF) was 23.9 ± 8.2%, mean end-diastolic volume was 195 ± 67 ml/m2. The study was supported by grants from Delegation à la recherche clinique AP-HP (EMUL and PHRC n°AOM95082).

**EUROGENE study (EHF).** All cases ([5](#ref-duboscq-bidot_mutations_2009)) were patients of European origin (all born in Europe, from parents and grand-parents born in France or neighboring countries) with a diagnosis of idiopathic DCM, i.e. left ventricle end-diastolic volume/diameter >117% of predicted value according to age and body surface area on echocardiography and low ejection fraction (<45%) confirmed over a three-month period, in the absence of causal factors such as coronary artery disease (coronary angiography or coronary CT scan was mandatory if DCM occurred after 35 years of age) or intrinsic valvular disease, documented myocarditis, systemic disease, sustained rapid supraventricular arrhythmia or congenital malformation. Recruitment was performed in 11 hospitals in seven European countries from September 2000 to February 2005. Only patients from France (3 centers in Paris-Ile de France), Italy (1 center in Pavia) and Germany (3 centers in Regensburg, Marburg and Munster) were selected for the study. A total of 392 DCM cases were included (311 men and 81 women, only 1 patient had undergone a cardiac transplantation at inclusion). Mean age of patients at diagnosis was 46.7 ± 12.7 years, mean LVEF was 29.2 ± 10.5%, mean end-diastolic diameter was 68 ± 10 mm. The study was supported by grants from the “Fondation LEDUCQ”. Control subjects for Italian DCM cases were selected from healthy consultants or hospital professional workers in clinical centers in Italy (93 controls, 71 men and 22 women).

**PHRC study.** DCM cases were French patients of European origin (all born in France, from parents and grand-parents born in France or neighboring countries; some patients of Maghreb origin were retrospectively excluded) with a diagnosis of idiopathic DCM (enlarged left ventricle end-diastolic volume/diameter >117% of predicted value according to age and body surface area on echocardiography and low ejection fraction (<45%) clinically stable over a three-month period, in the absence of causal factors such as coronary artery disease (coronary angiography or coronary CT scan was mandatory if DCM occurred after 35 years of age) or intrinsic valvular disease, documented myocarditis, or congenital malformation. Only apparently sporadic DCM cases without additional (first degree) relative with DCM were included. Recruitment was performed in eight hospitals in six regions in France (Lille, Lyon, Nantes, Nice, Paris-Ile de France, and Tours) from October 2005 to November 2008. A total of 207 DCM cases were included (166 men and 41 women, no patients had undergone a cardiac transplantation at inclusion). Mean age of patients at diagnosis was 52.2 ± 13.3 years, mean LVEF was 28.3 ± 9.1%, mean end-diastolic diameter was 69 ± 9 mm. The study was supported by grants from “Programme Hospitalier de Recherche Clinique” (PHRC n°AOM 04141).

**German study cohort.** German idiopathic DCM cases were recruited at the German Heart Institute Berlin and were of white European origin. Inclusion criteria for DCM cases were the following: reduced systolic function (LVEF <45%), after exclusion of major coronary artery disease (by angiography), significant (>grade 2) valvular heart disease, hypertensive heart disease, congenital heart disease, myocarditis or other secondary forms of heart failure. Patients with a positive family history were also excluded. A total of 972 DCM patients were included (808 men and 164 women). Mean age of patients at diagnosis was 43.1 ± 11.6 years, mean LVEF was 24.1 ± 9.7%.

**Boston study population.** USA 1 population. Boston subjects were recruited as research study subjects from numerous heart failure clinics throughout the US. Inclusion criteria for the Boston subjects were as follows: European descent, diagnosis of DCM, both idiopathic sporadic DCM as well as familial DCM, with low ejection fraction (<45%) and enlarged left ventricle end-diastolic volume/diameter >117% of predicted value according to age and body surface area on echocardiography. Exclusion criteria included: causal factors such as significant coronary artery disease or intrinsic valvular disease, documented myocarditis, systemic disease, or sustained arterial hypertension (sufficient per se to explain DCM), or congenital malformation. A total of 136 DCM cases were included (87 men and 49 women, including 24 cardiac transplant cases). Mean age of patients at diagnosis was 40.5 ± 14, mean LVEF was 29.4 ± 13.6. Control subjects were matched for age, gender and Caucasian origin (192 controls, 44 men and 148 women).

**UK study population.** Patients referred to the Royal Brompton and Harefield Hospitals NHS Foundation Trust (RBHT) cardiovascular magnetic resonance (CMR) unit from July 2001 to August 2012 for evaluation of a possible diagnosis of DCM and who agreed to provide samples for biobanking were prospectively recruited at the National Institute for Health Research Cardiovascular Biomedical Research Unit, RBHT and Imperial College London. Referrals were from centers across Southern England. A diagnosis of DCM was confirmed, and evaluated against published CMR criteria (ejection fraction >2sd below and end diastolic volume >2sd above the mean normalized for age and sex) by two independent Level 3 accredited CMR cardiologists. No patients had clinical symptoms or signs of active myocarditis or CMR evidence of infiltrative disease. Significant coronary artery disease (CAD) (>50% diameter luminal stenosis in any coronary artery) was excluded by coronary angiography. A total of 101 DCM cases were included (82 men and 19 women). Mean age of patients at diagnosis was 54.9 ± 13.4, mean LVEF was 30 ± 8.7. Control subjects were matched for age and gender (96 controls, 76 men and 20 women).

**PPS3 study.** The 4056 French controls (2515 men and 1541 women) were selected from the Paris Prospective Study III (PPS3), which is an ongoing French prospective study. From June 2008 to May 2012, 10157 men and women aged 50-75 years who had a preventive medical check-up at the Centre d’Investigations Préventives et Cliniques in Paris, were enrolled in the PPS3 study, after signing an informed consent. A detailed study report is described elsewhere.(6)

**KORA F4.** The 2921 German controls (1415 men and 1506 women) were selected from the KORA F4 study (Cooperative Health Research in the Augsburg Region) which is a 7-year follow-up study to the population-based KORA Survey S4. The baseline survey KORA S4 was conducted in the years 1999–2001 in the city of Augsburg, Southern Germany, and its two adjacent counties. All survey participants are residents of German nationality identified through the registration office within the age range 25-74 years and gave written consent.

**MAGnet Study** USA 2 population. Subjects with dilated cardiomyopathy defined as patients with heart failure and an ejection fraction < 40% in the absence of hypertension, primary valvular disease, or coronary artery disease we recruited in the MAGNet Study. All subjects provided written informed consent using protocols approved by relevant institutional review boards. Whole genome SNP genotypes were generated in the Center for Applied Genomics (Philadelphia, USA), using the Illumina *HumanOmniExpressExome8v1_A BeadChip*. Genotypes calling was done using *GenomeStudio* v2011.1 and after quality control and limitation of the dataset to genetically inferred Caucasians, 631 DCM cases were kept in the study.

### Protein interaction study

**Reagents and antibodies.** Chemicals were purchased from Sigma (Sigma-Aldrich, St Louis, MO, USA). Antibody against α-tubulin (ab7291, Abcam, Cambridge, UK) was mouse monoclonal. Antibodies against EGFP (TP401, CliniSciences, Nanterre, France) and HspB7 (15700-1-AP, ProteinTech Group, Chicago, IL, USA) were rabbit polyclonal. Plasmids construction: All constructs were validated by sequencing after cloning. For GST pull-down, the cDNA of HSPB7 was PCR amplified from human heart mRNA after reverse transcription with primers (HspB7 Fw 5’-AGCGAATTCATGAGCCACAGAACC-3’ and HspB7 Rev 5’-GCGCTCGAGTCAGATTTTGAT-CTCCG-3’) and cloned in pCR2.1 using the TA cloning kit (Life Technologies) according to the manufacturer’s protocol. The cDNA was sub-cloned from pCR2.1-HspB7 into pGEX-6P-1 (GE Healthcare, Uppsala, Sweden), in frame with the glutathione S-transferase (GST) using BamHI and NotI restriction enzymes (NEB) in order to obtain the pGEX-HSPB7 plasmid. For immunoprecipitation, the cDNA of HSPB7 was PCR amplified from human heart mRNA after reverse transcription with primers with primers (HspB7-ΔSTOP Fw 5’-ACTTGGATCCATGAGCCACAGAACCTCTTCCA-3’ and HspB7-ΔSTOP Rev 5’-CATTAAGCTT-GATTTTGATCTCCGTCCGGAA-3’) in order to remove the STOP codon from HspB7. The cDNA was cloned in pCR2.1 using the TA cloning kit (Life Technologies) according to the manufacturer’s protocol and then sub-cloned into pCMV-3Flag-3A plasmid (Agilent Technologies), in frame with the 3 Flag, generating the pCMV-HSPB7-3Flag vector.

**Cell culture, transfection.** HEK293 (human embryonal kidney) cells were grown in Dulbecco’s modified Eagle’s medium (Invitrogen, Carlsbad, CA, USA) supplemented with 10% fetal bovine serum and 1% penicillin/streptomycin (Invitrogen, Carlsbad, CA, USA) at 37°C under a humidified atmosphere containing 5% CO2. HEK293 were transfected using JetPEI (Polyplus, Illkirch, France) according to the manufacturer’s instructions with 8µg of total plasmid DNA per 75cm2 flask. HEK293 cells were transfected with 8µg of pEGFP-BAG3 or pEGFP-N3 for GST pull-down experiments, or cells were co-transfected with pEGFP-BAG3 (kind gift from Professor S. Takayama) or pEGFP-N3 (Clontech), and pCMV-HSPB7-3Flag for co-immunoprecipitation experiments. 48 hours later, cells were harvested for biochemical interaction experiments.

**Protein extraction.** Cells were washed with PBS and lysed in the lysis buffer A (Tris pH 7.5 50mM, NaCl 150mM, EDTA 2mM, sodium orthovanadate 2mM, PMSF 1mM and complete protease inhibitor cocktail from Sigma-Aldrich). Cell pellets were flushed 5 times through a 22-gauge needle, rotated for 1 h at 4°C, and finally centrifuged for 15 min, 11000 rpm, 4°C. The supernatants were used for the experiments.

**Co-immunoprecipitation.** Total cell extracts were pre-cleared with magnetic Protein A Dynabeads (Dynal, Norway) for 1 h at 4°C. The unbound extracts were then incubated with the anti-GFP antibody for 2h. Dynabeads were washed twice with PBS-tween 0.02% and incubated with the protein-antibody complexes. Samples were rotated overnight at 4°C. After washing the beads 4 times with PBS-tween 0.02%, proteins were eluted with the BPP2X buffer (Bromophenol Blue 1%, glycerol 50%, SDS 20%, DTT 20mM) at 95°C for 10 min under agitation. Both co-immunoprecipitated proteins and input fractions were resolved by SDS-PAGE.

**GST pull-down.** GST tagged HspB7 proteins were obtained by transformation of the pGST-HSPB7 plasmid into BL21-DE3 *E.Coli* cells (Life Technologies). The next day, a single colony was picked to inoculate a 5mL overnight culture at 225rpm, 37°C of YTA 2X medium (tryptone 16g/L, yeast extract 10g/L, NaCl 5g/L, pH7). Then, 1mL from the overnight culture was added to 250mL of YTA 2X medium and grown to an A600 of 0.6-0.8 (225rpm, 37°C). To induce the fusion protein expression, 1mM IPTG was added to the culture which incubated for an additional 1-2h (225rpm, 30°C). Cells were harvested and centrifuged for 20min, 9500rpm, 4°C. The cell pellet was lysed using the lysis buffer B (Tris pH7.5 40mM, NaCl 500mM, NP40 1%, EDTA 2mM, PMSF 1%) supplemented with lysozyme (10mg/mL). After sonication and solubilization with Triton 1%, soluble fraction was collected (centrifugation for 20min, 9500rpm, 4°C). The GST-HspB7 proteins were complexed to Glutathione Sepharose 4B beads (GE Healthcare) by a 6H incubation on a rotor at 4°C.
Total HEK 293 cell extracts were pre-cleared with Glutathione Sepharose 4B beads for 2 h at 4°C under rotation. The unbound proteins were rotated with GST-HspB7 proteins overnight at 4°C. After washing the beads 4 times with the lysis buffer B, protein were eluted with the BPP2X buffer at 95°C for 10 min under agitation. All centrifugations were performed for 2 min, 1000 rpm, 4°C. Both GST pull-down proteins and input fractions were resolved by SDS-PAGE.

**Western-Blot.** Samples were prepared in BPP2X buffer and boiled at 95°C, 10min before loading. Equal amount of proteins were loaded and separated on 10 or 12% acrylamide SDS-PAGE gels and, following electrophoresis, were transferred to nitrocellulose membranes (iBlot® Transfert Stack, IB3010-31, Life Technologies) using an iBlot® Gel Transfert device (Life Technologies). They were then blocked for two hours in 5% non-fat milk in PBS with 0.1% Tween. Primary antibodies were used at the following dilutions: EGFP (1:1000), α-tubulin (1:1250), HspB7 (1:800). Blots were incubated with primary antibodies overnight at 4°C, rinsed and incubated 1 hour with secondary antibodies conjugated to the infra-red dyes 680LT or 800CW (LI-COR® Biosciences, NE, USA) at 1:10000 dilution. Blots were imaged using the Odyssey® Imager (LI-COR® Biosciences). Protein signal intensities were observed with the ImageJ freeware (version 1.41, NIH, Bethesda, MD, USA, http://rsb.info.nih.gov/ij/).

### Genotyping

Each DNA sample was quality checked on agarose gel and quantified by QUANT-IT Picogreen dsDNA reagent (*Invitrogen*). Concentration adjustment and aliquoting of samples was automatized on a robot (*Beckman Biomek 3000*) to reduce technical variability. For genotyping we used the *Illumina HumanExome-12v1.1 BeadChip* and applied the standard *Infinium ® HD Assay Ultra* protocol, automated according to manufacturer instructions. Briefly 200 ng DNA per sample was amplified, fragmented and hybridized to the *BeadChips*. The extension/staining of DNA was followed by imaging of BeadChips with the *iScan system* 110 V/220 V (*Illumina*). Samples were genotyped on the P3S platform (Paris 6 University and INSERM, Paris, France). Genotyping of 2,921 individuals of the KORA F4 cohort was performed at the Helmholtz Zentrum (Munich, Germany) using the *Illumina HumanExome-12v1_A BeadChip*. In comparison to the v1.1 version, this array harbors 4,969 additional markers. Genotypes generated by both arrays were separately called and only variants present on both arrays were considered for analyses. Genotype calling was carried out using *Illumina’s GenTrain* version 2.0 clustering algorithm and genotyping module version 1.9.4 in *GenomeStudio* v2011.1. The *humanexome-12v1.egt* and *humanexome-12v1-1_a.egt* cluster files were used respectively for each version of the array and the no-call threshold was set to 0.15. The re-clustering option provided by *GenomeStudio* was applied for all variants having at least one no-call. The quality of genotypes clustering was visually checked *a posteriori* for all variants associated with DCM. Genotyping of the 631 DCM patients from the MAGNet study (USA2) was done at the Center for Applied Genomics (Philadelphia, USA), using the Illumina *HumanOmniExpressExome8v1_A BeadChip* and genotypes calling was done using *GenomeStudio* v2011.1.

### Data preprocessing

#### Quality control

Quality control was performed with the 1.9 version of the *PLINK* software(7) and in the *R* version 3.1 environment([8](#ref-team_language_2013)). Markers with genotyping success rate < 99% (n = 1412 variants) and samples with < 99% of markers available (n = 143 samples) were excluded. Within each population, markers not in Hardy-Weinberg equilibrium in controls (P < 10^−5^) and outlier samples identified using the "neighbor" function implemented in *PLINK* were not investigated further (n = 122). We used the *Genome-wide complex trait analysis* software (*GCTA*)([9](#ref-yang_genome_2013)) to detect cryptic relatedness among study participants and removed one individual from each pair of related samples using a grm-cutoff = 0.1 (n = 186). To minimize the consequences of using a different version of the exome array in the German control group, we assessed differential missingness (unsuccessful genotyping) between the French and German control groups and excluded variants whose missingness differed between the 2 groups (P < 10^-5^, n = 754 variants). For loci that included multiple variants on the exome array that were in linkage disequilibrium (LD), we analyzed one variant in each pair of nearby variants with high LD (the window size, step size and R2 threshold parameters in *PLINK* "--indep-pairwise" were set at 100kb, 10 and 0.5 respectively). This procedure excluded 9825 variants.

#### Variant filtering does not affect the results significantly

Due to the design of the exome array which at many loci includes several coding variants that are in LD, it was decided at the data pre-processing stage to exclude one variant from each pair exhibiting high LD (see material and methods). This led to the exclusion of 9828 variants. To ensure that this filtering did not exclude important variants, we checked the association of the excluded variants with DCM using the same approach as for the variants kept in the analysis. Four among the excluded variants were significantly associated with DCM: a missense variant in the *SEC23IP* gene (rs2475298 at position 10:121679013, P = 3.89x10^-08^), 2 missense variants in the *TTN* gene (rs2042996 at position 2:179451420, P = 3.34x10^-07^ and rs9808377 at position 2:179421694, P = 4.25x10^-07^) and a variant in the 5'UTR of *HSPB7* (rs872222 at position 1:16344664, P = 8.61x10^-07^). *SEC23IP* is located close to *BAG3* and after adjustment on the respective lead-SNVs in *BAG3*, TTN* and *ZBTB17* the association of the filtered out SNVs with DCM was no longer significant. We therefore conclude that the filtering applied to variants in LD did not mask any important locus associated with DCM.

### Statistical analysis

#### Variant-level analysis.

Association between case-control status and each variant was assessed using logistic regression, variant effect being modeled either as additive or dominant in *PLINK*. To account for a possible population stratification, the 20 first principal components (PCs) estimated from the genetic relatedness matrix (GRM) computed from all available autosomal variants using GCTA(9) were included in the logistic model. To assess the homogeneity of effects of DCM-associated variants across populations, we analyzed population-specific data sets separately using logistic regression adjusted on the first 20 population-specific PCs and summarized the effect estimates in a meta-analysis with fixed-effects. Population-specific odd-ratios estimates and their 95% CI are displayed in Forest plots (R/rmeta package). For the within population analysis, a control group was artificially defined for the patients in the MAGNet Study (USA2) by subsampling 1,000 individuals from the German control group. The remaining 1,830 German control subjects were used as controls for the German cases. Based on the QQ-plot (***S2 Fig.***) and lambda value (0.969) for the USA2 cohort, the reconstructed control group for the MAGNet DCM cases was appropriate. Associations are summarized in a Manhattan plot (R/qqman package) and the fit of observed to expected association statistics is plotted in a QQ-plot (R/snvStats package). The lambda value was used to assess the fit of the observed distribution of association chi-square statistics to that expected under no association. To account for multiple testing, a Q-value (R/Qvalue package) threshold of 0.01 was chosen. "The Q-value is similar to the well-known P-value, except it is a measure of significance in terms of the false discovery rate rather than the false positive rate". ([10](#ref-storey_statistical_2003))

#### Candidate gene and DCM gene set analyses

At the gene and gene-set levels, associations traits were investigated using the Sequence Kernel Association Tests (SKAT)(11) implemented in the R package *SKAT* for both rare and common variants (*SKAT* "CommonRare" function). All tests were adjusted on age, gender and first 20 PCs by entering these covariates in the SKAT null model. The *SKAT* test allows for both effect-increasing and effect-decreasing variants, this appears appropriate in the context of this study in which tested variants were collected independently of the phenotypes investigated. The gene-level analysis was performed using the *R/SKAT_CommonRare* function. We used the default values of the parameters in *SKAT*, in particular the beta-weights were set at [1,25] for rare variants and [0.5,0.5] for common ones. For each variant, the region covered corresponds to that displayed in ***Fig. 3*** (regional plots), *ie.* start/end gene sequence -/+ 100KB, except for *BAG3* and *ALPK3* to cover the regional LD block. Note that all variants available were included in the skat analyzes, including rare ones which were not tested in the variant-level analysis because they were either absent from the case or from the control groups. The adjusted P-values were obtained with *SKAT* for each region by excluding the lead SNP from the tested variant set and including it in the covariables set for adjustment.

For the DCM gene-set analysis, all variants on 48 genes that have been reported to be associated with Familial DCM (excluding *BAG3* and *TTN*) that were available in the data set were analyzed with *SKAT*. Subset of variants categorized by severity according to *CADD* were also investigated. Reference genome SNVs at the 10th-% of *CADD* scores are assigned to *CADD*-10, top 1% to *CADD*-20, top 0.1% to *CADD*-30, etc. (http://cadd.gs.washington.edu/info).

#### Imputation of variants in the regions of interest

The objective was to identify non-genotyped variants that are in strong LD with the DCM-associated lead-SNVs. Imputation was conducted across regions encompassing the sequence of the best candidate genes identified in the variant-level analysis and 500KB (750KB for *ALPK3*) upstream and downstream. Within these regions, haplotypes were estimated using the phasing program *SHAPEIT*([12](#ref-delaneau_improved_2013)) and unmeasured genotypes were imputed with *IMPUTE2* version 2.3.2([13](#ref-howie_flexible_2009)) using the last release of the *1000 genomes* haplotypes database (Phase 3 integrated variant set, updated 12 Oct 2014), which is based on 2504 samples of multi ethnic origin. The complete *1000 genomes* data set was used for imputation as advised by the authors of *IMPUTE2* [https://mathgen.stats.ox.ac.uk/impute/impute_v2.html#using_multipop_panels]. To reduce the possibility of biased imputation, only variants imputed in at least 80% of samples with a quality > 0.90 (*impute 2* 'info' metrics) for which missing genotype frequencies did not differ between cases and controls (P > 0.01) were considered. In ***S3*** ***Table*** the number of subjects included in the case-control comparisons and the value of the *IMPUTE2* 'info' metric ("Quality") are provided. For each imputed variant passing QC, logistic regression analysis adjusted on the first 20 PCs was conducted as for genotyped variants (see "variant-level analysis").

#### Functional annotation of genes

To functionally assess coding as well as non-coding variants we used the *Combined Annotation Dependent Depletion* (*CADD*) framework. *CADD* "integrates multiple annotations into one metric by contrasting variants that survived natural selection with simulated mutations".([14](#ref-kircher_general_2014)) The *CADD* scores reported in ***S3*** ***Table*** rank variants in increasing order of putative severity from 0 to 100.

#### Tissue specific expression

We assessed tissue-specific gene expression *in silico* using the Genotype-Tissue Expression (*GTEx*) project database(15). This resource provides expression and eQTL information in a large series of tissues, including "Heart Left Ventricle" (87 samples), heart atrial appendage (27 samples) and "Skeletal Muscle" (143 samples) (http://www.gtexportal.org/home/).

#### Exon usage in TTN, missense variants and DCM risk

Truncating (TTNtv) and missense variants of *TTN* are a common cause of DCM ([16](#ref-gerull_mutations_2002),[17](#ref-herman_truncations_2012)), however such variants are common in the general population suggesting that most of them have low or no penetrance.([17](#ref-herman_truncations_2012)) It is known that the proportion of transcripts that incorporate a given exon (PSI) is a useful index to discriminate between pathogenic and tolerated TTNtv. In DCM patients TTNtv are enriched in exons with high PSI, whereas TTNtv in healthy individuals frequently occur in exons that are spliced out of expressed transcripts (low PSI).(18) To see whether this could also be true for *TTN* missense variants present in our data (the exome array contained no TTNtv), we categorized these variants by their PSI scores (<0.9 vs. >=0.9). *SKAT* analysis indicated that in both groups, rare and common variants are similarly associated with DCM (Low PSI: common (n=119), P < 0.0077; rare (n=23), P < 1.25 x 10^-5^); High PSI: common (n=237), P < 0.020; rare (n=59), P < 2.70 x 10^-5^). This suggests that the usage of *TTN* exons containing missense variants present on the exome-array does not differ between DCM patients and controls

### Power Analysis.

Common variants were analyzed at the variant level (S1a Table) and rare variants were analyzed at the gene/region/set levels (S1b Table).


**Gene/region/set levels power analysis**. For sets of variants, power estimation requires modeling the genetic architecture (variants number, respective frequencies and effects, LD between variants). The R/SKAT package (version 1.0.7) provides an analytic method to compute power for SKAT. The approach makes use of a simulated data set of 10,000 haplotypes, encompassing 3845 variants (3266 with MAF < 0.01) over a 200k BP region, generated according to a coalescent model that mimics the LD pattern, local recombination rate and population history of Europeans. Instead of directly specifying the variant effects, the MAF threshold for rare variants, proportion of causal variants among rare variants, fraction of rare variants negatively associated with the phenotype, and the effect size are specified by the user. Within this framework, effect size may be modeled either as constant or as being inversely related to MAF. Power and sample size are estimated by averaging results over a range of parameter values and to account for the heterogeneity of genetic architecture, results are averaged across a number (N.Sim) of randomly selected sub-regions whose size (number of variants) may be set to various values that are relevant for the conducted study.


## Supplementary References

1. Elliott P, Andersson B, Arbustini E, Bilinska Z, Cecchi F, Charron P, Dubourg O, Kühl U, Maisch B, McKenna WJ, Monserrat L, Pankuweit S, Rapezzi C, Seferovic P, Tavazzi L, Keren A. Classification of the cardiomyopathies: a position statement from the European Society Of Cardiology Working Group on Myocardial and Pericardial Diseases. *European Heart Journal*. 2008;**29**:270–276.

2. Henry WL, Gardin JM, Ware JH. Echocardiographic measurements in normal subjects from infancy to old age. *Circulation*. 1980;**62**:1054–1061.

3. Mestroni L, Maisch B, McKenna WJ, Schwartz K, Charron P, Rocco C, Tesson F, Richter A, Wilke A, Komajda M. Guidelines for the study of familial dilated cardiomyopathies. Collaborative Research Group of the European Human and Capital Mobility Project on Familial Dilated Cardiomyopathy. *European Heart Journal*. 1999;**20**:93–102.

4. Charron P, Tesson F, Poirier O, Nicaud V, Peuchmaurd M, Tiret L, Cambien F, Amouyel P, Dubourg O, Bouhour J, Millaire A, Juilliere Y, Bareiss P, André-Fouët X, Pouillart F, Arveiler D, Ferrières J, Dorent R, Roizès G, Schwartz K, Desnos M, Komajda M. Identification of a genetic risk factor for idiopathic dilated cardiomyopathy. Involvement of a polymorphism in the endothelin receptor type A gene. CARDIGENE group. *European Heart Journal*. 1999;**20**:1587–1591.

5. Duboscq-Bidot L, Charron P, Ruppert V, Fauchier L, Richter A, Tavazzi L, Arbustini E, Wichter T, Maisch B, Komajda M, Isnard R, Villard E, EUROGENE Heart Failure Network. Mutations in the ANKRD1 gene encoding CARP are responsible for human dilated cardiomyopathy. *European Heart Journal*. 2009;**30**:2128–2136.

6. Empana J-P, Bean K, Guibout C, Thomas F, Bingham A, Pannier B, Boutouyrie P, Jouven X, PPS3 Study Group. Paris Prospective Study III: a study of novel heart rate parameters, baroreflex sensitivity and risk of sudden death. *European Journal of Epidemiology*. 2011;**26**:887–892.

7. Purcell S, Neale B, Todd-Brown K, Thomas L, Ferreira MAR, Bender D, Maller J, Sklar P, Bakker PIW de, Daly MJ, Sham PC. PLINK a tool set for whole-genome association and population-based linkage analyses. *American journal of human genetics*. 2007;**81**:559–575.

8. Team RC. R A Language and Environment for Statistical Computing [Internet]. Vienna, Austria: R Foundation for Statistical Computing; 2013. Available from: <http://www.R-project.org/>

9. Yang J, Lee SH, Goddard ME, Visscher PM. Genome wide complex trait analysis (GCTA): methods, data analyses, and interpretations. *Methods in molecular biology (Clifton, NJ)*. 2013;**1019**:215–236.

10. Storey JD, Tibshirani R. Statistical significance for genomewide studies. *Proceedings of the National Academy of Sciences of the United States of America*. 2003;**100**:9440–9445.

11. Wu MC, Lee S, Cai T, Li Y, Boehnke M, Lin X. Rare-Variant Association Testing for Sequencing Data with the Sequence Kernel Association Test. *The American Journal of Human Genetics*. 2011;**89**:82–93.

12. Delaneau O, Zagury J-F, Marchini J. Improved whole-chromosome phasing for disease and population genetic studies. *Nature Methods*. 2013;**10**:5–6.

13. Howie BN, Donnelly P, Marchini J. A Flexible and Accurate Genotype Imputation Method for the Next Generation of Genome-Wide Association Studies. *PLoS Genetics*. 2009;**5**.

14. Kircher M, Witten DM, Jain P, O’Roak BJ, Cooper GM, Shendure J. A general framework for estimating the relative pathogenicity of human genetic variants. *Nature Genetics*. 2014;**46**:310–315.

15. GTEx Consortium. The Genotype-Tissue Expression (GTEx) project. *Nature Genetics*. 2013;**45**:580–585.

16. Gerull B, Gramlich M, Atherton J, McNabb M, Trombitás K, Sasse-Klaassen S, Seidman JG, Seidman C, Granzier H, Labeit S, Frenneaux M, Thierfelder L. Mutations of TTN, encoding the giant muscle filament titin, cause familial dilated cardiomyopathy. *Nature Genetics*. 2002;**30**:201–204.

17. Herman DS, Lam L, Taylor MRG, Wang L, Teekakirikul P, Christodoulou D, Conner L, DePalma SR, McDonough B, Sparks E, Teodorescu DL, Cirino AL, Banner NR, Pennell DJ, Graw S, Merlo M, Di Lenarda A, Sinagra G, Bos JM, Ackerman MJ, Mitchell RN, Murry CE, Lakdawala NK, Ho CY, Barton PJR, Cook SA, Mestroni L, Seidman JG, Seidman CE. Truncations of titin causing dilated cardiomyopathy. *The New England Journal of Medicine*. 2012;**366**:619–628.

18. Roberts AM, Ware JS, Herman DS, Schafer S, Baksi J, Bick AG, Buchan RJ, Walsh R, John S, Wilkinson S, Mazzarotto F, Felkin LE, Gong S, MacArthur JAL, Cunningham F, Flannick J, Gabriel SB, Altshuler DM, Macdonald PS, Heinig M, Keogh AM, Hayward CS, Banner NR, Pennell DJ, O’Regan DP, San TR, Marvao A de, Dawes TJW, Gulati A, Birks EJ, et al. Integrated allelic, transcriptional, and phenomic dissection of the cardiac effects of titin truncations in health and disease. *Science Translational Medicine*. 2015;**7**:270ra6.
