# Supplementary material for: Exome-wide association study reveals novel susceptibility genes to sporadic dilated cardiomyopathy
Source: PLoS One. 2017 Mar 15;12(3):e0172995. doi: 10.1371/journal.pone.0172995 (PMC5351854; doi:10.1371/journal.pone.0172995)

Figure S1. QQ Plot of association P-values.

The lambda value, which assess the fit of the observed distribution of association chi-square statistics to that expected under no association, was 0.991.

note: The plots in figures S1 and S2 are based on the P values obtained by logistic regression analysis, using the function qq.chisq in the R package snpStats. To account for the fact that the tests may have 1 or 2 df, -2log(P) values are plotted as chi-squared on 2 df as suggested in the vignette of R/snpStats.

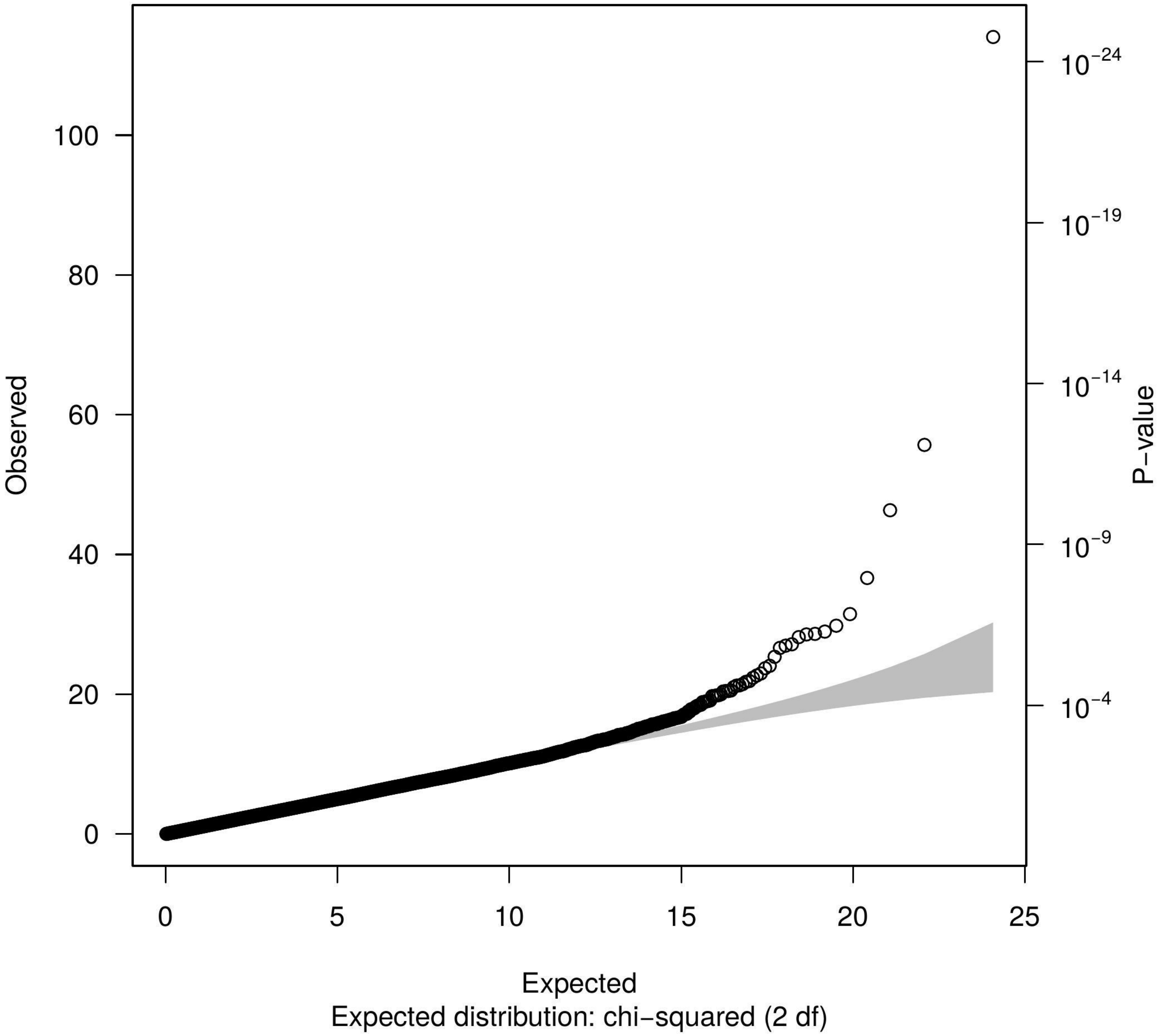

Supplement: S1 Fig — (PDF) [file pone.0172995.s002.pdf]
