# Supplementary material for: Exome-wide association study reveals novel susceptibility genes to sporadic dilated cardiomyopathy
Source: PLoS One. 2017 Mar 15;12(3):e0172995. doi: 10.1371/journal.pone.0172995 (PMC5351854; doi:10.1371/journal.pone.0172995)

Figure S2. Manhattan and QQ plots by population

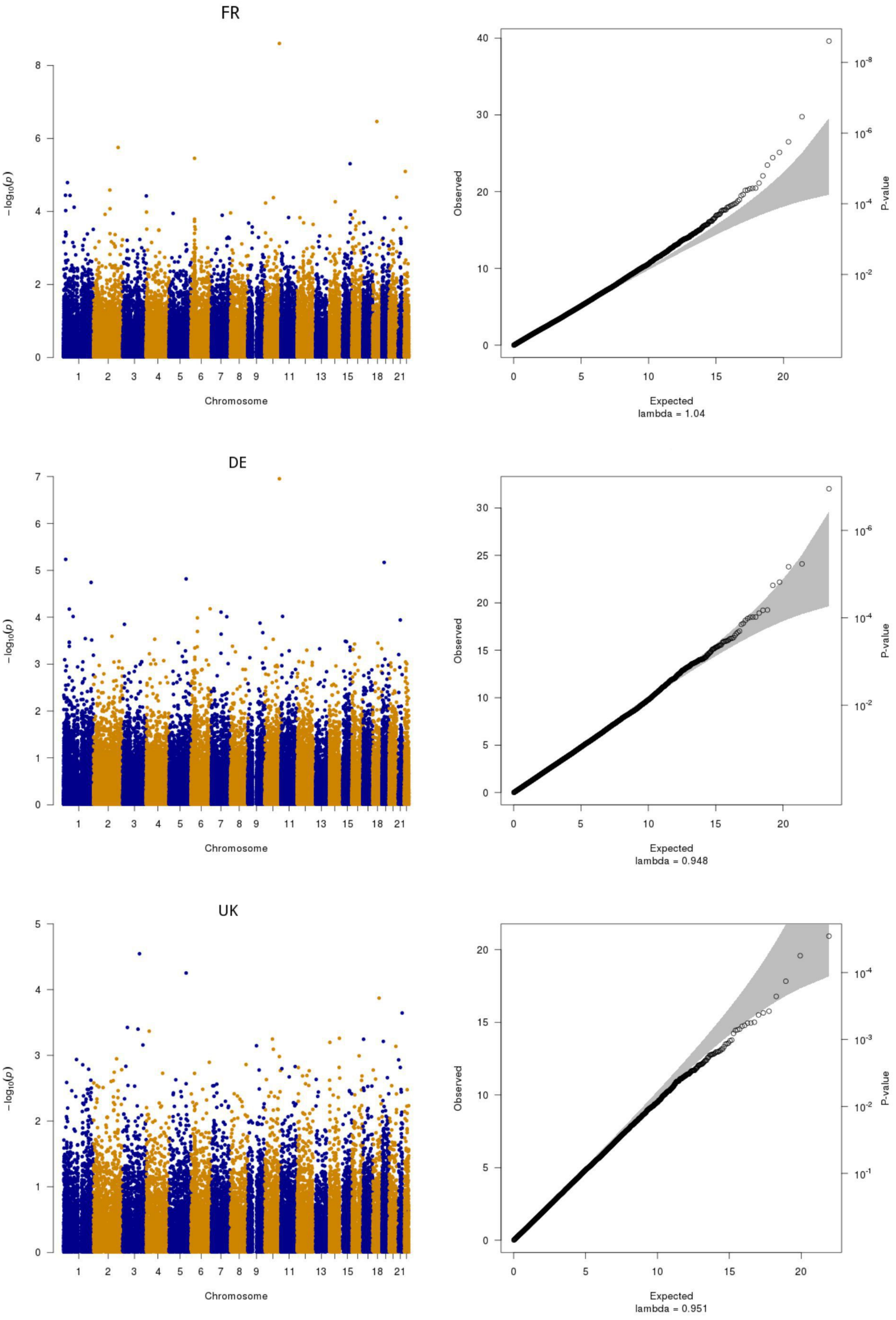

Supplementary figure 2. Manhattan and QQ plots by population. (cntd)

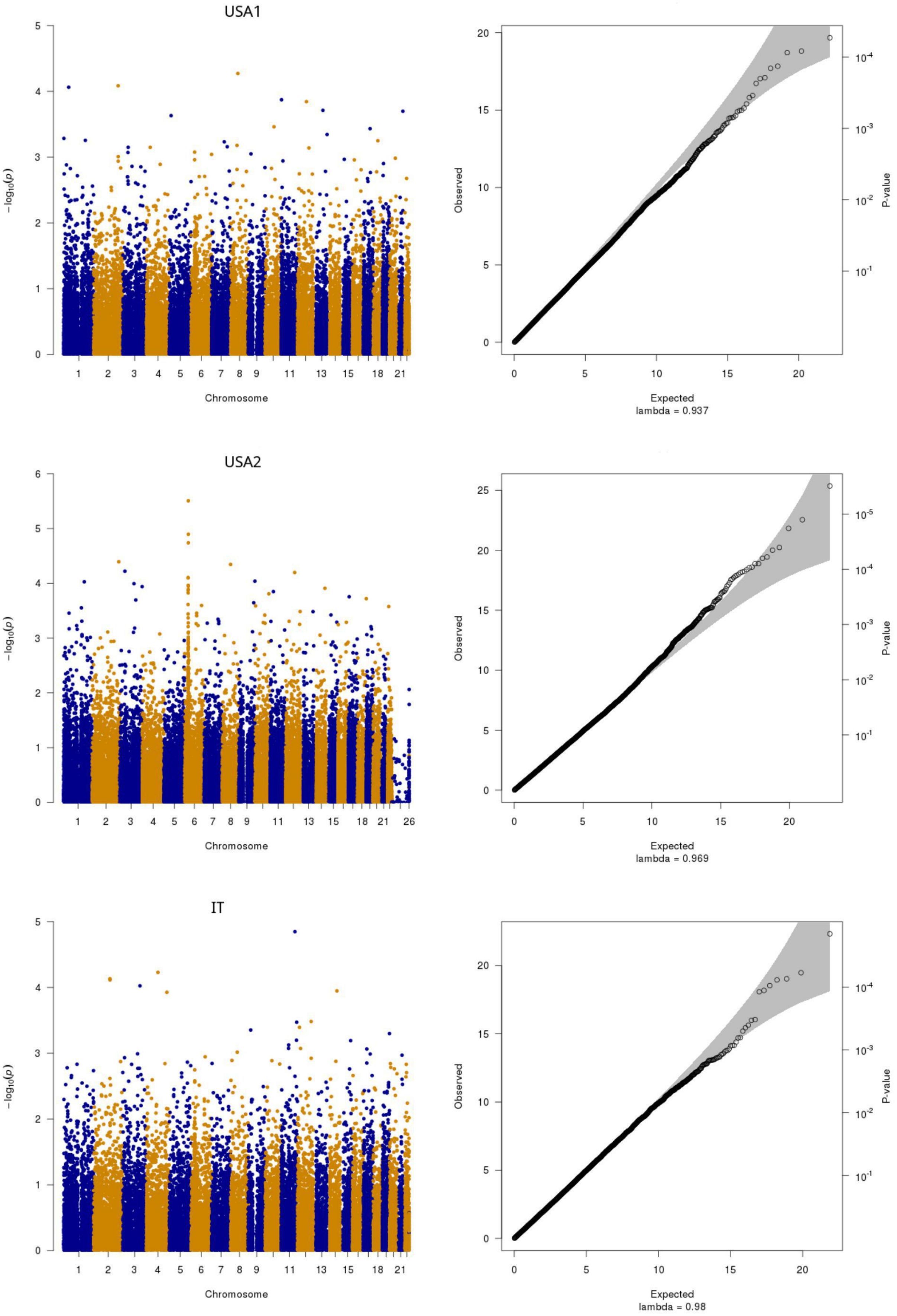

Supplement: S2 Fig — (PDF) [file pone.0172995.s003.pdf]
