# Supplementary material for: Exome-wide association study reveals novel susceptibility genes to sporadic dilated cardiomyopathy
Source: PLoS One. 2017 Mar 15;12(3):e0172995. doi: 10.1371/journal.pone.0172995 (PMC5351854; doi:10.1371/journal.pone.0172995)
Supplement: S1 Table — (DOCX) [file pone.0172995.s005.docx]

##### S1a Table. Power of the study to detect effect of single common variants.

| Frequency | RR=1.25 | RR=1.5 | RR=1.75 | RR=2.0 |
| --- | --- | --- | --- | --- |
| 0.010 | <0.1 | <0.1 | 0.21 | 0.67 |
| 0.025 | <0.1 | 0.32 | >0.9 | >0.9 |
| 0.050 | <0.1 | >0.9 | >0.9 | >0.9 |
| 0.100 | 0.29 | >0.9 | >0.9 | >0.9 |
| 0.250 | 0.90 | >0.9 | >0.9 | >0.9 |

***S1a Table*** reports the power of our study for various MAF and allelic risk. The computations were done with the Genetic Power Calculator (Case-control for discrete traits) ([Purcell](#ref-purcell_genetic_2003) et al, Bioinformatics, 2003:19:149-150) using the following parameters:

- High risk allele frequencies: 0.01, 0.1 and 0.25
- Prevalence: 0.002
- allelic relative risk: 1.25, 1.5, 2.0
- D-prime = 1 and marker allele frequency = High risk allele frequencies
- Number of cases: 2796
- Control : case ratio: 2.46
- type I error: 5 x 10^-7^

##### S1b Table. Power of the study to detect effect of set of rare variants.

| causal | RR | alpha | nvar=25 | nvar=50 | nvar=75 | nvar=100 | nvar=500 |
| --- | --- | --- | --- | --- | --- | --- | --- |
| 5% | 3 | 0.00625 | <0.1 | <0.1 | <0.1 | <0.1 | 0.32 |
| 5% | 3 | 4x10^-6^ | <0.1 | <0.1 | <0.1 | <0.1 | 0.10 |
| 5% | 5 | 0.00625 | <0.1 | 0.16 | 0.19 | 0.24 | 0.75 |
| 5% | 5 | 4x10^-6^ | <0.1 | <0.1 | <0.1 | <0.1 | 0.43 |
| 5% | 10 | 0.00625 | 0.26 | 0.44 | 0.51 | 0.62 | 0.94 |
| 5% | 10 | 4x10^-6^ | 0.11 | 0.23 | 0.27 | 0.29 | 0.79 |
| 10% | 3 | 0.00625 | <0.1 | 0.10 | <0.15 | <0.25 | 0.59 |
| 10% | 3 | 4x10^-6^ | <0.1 | <0.1 | <0.1 | <0.1 | 0.25 |
| 10% | 5 | 0.00625 | 0.21 | 0.35 | 0.41 | 0.50 | >0.9 |
| 10% | 5 | 4x10^-6^ | <0.1 | 0.14 | 0.16 | 0.22 | 0.71 |
| 10% | 10 | 0.00625 | 0.51 | 0.71 | 0.81 | 0.88 | >0.9 |
| 10% | 10 | 4x10^-6^ | 0.26 | 0.43 | 0.54 | 0.62 | >0.9 |
| 25% | 3 | 0.00625 | 0.22 | 0.33 | 0.45 | 0.55 | >0.9 |
| 25% | 3 | 4x10^-6^ | <0.1 | <0.1 | 0.13 | 0.20 | >0.9 |
| 25% | 5 | 0.00625 | 0.52 | 0.75 | 0.86 | 0.90 | >0.9 |
| 25% | 5 | 4x10^-6^ | 0.26 | 0.40 | 0.56 | 0.62 | >0.9 |
| 25% | 10 | 0.00625 | 0.87 | >0.9 | >0.9 | >0.9 | 0.94 |
| 25% | 10 | 4x10^-6^ | 0.61 | 0.86 | 0.90 | >0.9 | >0.9 |

***S1b*** ***Table*** reports the power of our study for various number of variants (nvar), percentage of causal variants (%causal), allelic relative risk (RR) and type I error rate (alpha). The computations were done with R/SKAT using the following parameters (see R/SKAT 'Power_Logistic' function for more details on the parameters) :

- Number of variants: 25, 75, 50, 50, 100, 500 (values were chosen to reflect situations encountered in our study. Note that the number of variants is derived, it is the sub-region length which is provided as a parameter.)
- Disease Prevalence: 0.002
- allelic relative risk (MaxOR): 3, 5, 10
- Causal MAF cutoff (only variants with a MAF < cutoff are considered as causal): 0.01
- Causal Percent (Percentage of causal variants among rare variants): 5, 10, 25
- Number of cases/controls: 2796/6877
- type I error: 0.00625 and 4.16 x 10^-6^, corresponding respectively to a Bonferroni P-value of 0.05 corrected for the number of candidate regions (n = 8) and the number of genes (n = 12,000).
- Weight.Param: [1,25]
- Number of simulations (N.Sim): 200
- Negative.Percent: 0 (we neglect protective variants)

It appears that for sets including 25 variants or less, very high values of percentage of rare variants (> 25%) and allelic relative risk (> 10) would be needed to reach an acceptable power. When 50 variants are available (i.e. *ZBTB17*) and alpha = 0.00625, the power would reach 0.75 when 25% of the rare variants are causal and the maximum allelic RR = 5. When the variants set is large (nvar = 500, i.e. *TTN* or the DCM set) the power of the study is large under a number of conditions.
