# Supplementary material for: Exome-wide association study reveals novel susceptibility genes to sporadic dilated cardiomyopathy
Source: PLoS One. 2017 Mar 15;12(3):e0172995. doi: 10.1371/journal.pone.0172995 (PMC5351854; doi:10.1371/journal.pone.0172995)
Supplement: S2 Table — (DOCX) [file pone.0172995.s006.docx]

##### Table S2. Number of DCM patients and controls

|  | DCM (women) | CONTROLS (women) |
| --- | --- | --- |
| **France** | 706 (149) | 3677 (1394) |
| **Germany** | 1161 (205) | 1830 (959) |
| **Italy** | 83 (15) | 92 (23) |
| **UK** | 96 (20) | 89 (19) |
| **USA1** | 119 (43) | 189 (144) |
| **USA2** | 631 (211) | 1000 (506) |
| **All** | 2796 (643) | 6877 (3045) |

For the Meta-analysis, the USA2 control group was constituted of 1000 subjects randomly selected from the German control group, and the 1830 remaining German controls were used as controls for German DCM patients.
