# Supplementary material for: Exome-wide association study reveals novel susceptibility genes to sporadic dilated cardiomyopathy
Source: PLoS One. 2017 Mar 15;12(3):e0172995. doi: 10.1371/journal.pone.0172995 (PMC5351854; doi:10.1371/journal.pone.0172995)
Supplement: S3 Table — (DOCX) [file pone.0172995.s007.docx]

##### Table S3a. In Silico fine mapping at the *ZBTB17* locus (P < 10^-10^)

|  | Position | N | Quality | Ref/Alt | Gene | OR | P-value | Consequence | CADD score |
| --- | --- | --- | --- | --- | --- | --- | --- | --- | --- |
| **rs4661338** | 16131112 | 9028 | 0.953 | C/A | UQCRHL | 0.762 | 2.7x10^-11^ | downstream | 5.21 |
| **rs1976401** | 16143630 | 9142 | 0.963 | G/A | - | 0.785 | 9.5x10^-11^ | intergenic | 1.67 |
| **rs1976402** | 16143779 | 9358 | 0.973 | G/A | - | 0.757 | 1.4x10^-12^ | intergenic | 2.82 |
| **rs6674071** | 16144855 | 9333 | 0.973 | G/C | - | 0.761 | 3.7x10^-12^ | intergenic | 0.43 |
| **rs6699394** | 16145143 | 9146 | 0.966 | C/A | - | 0.785 | 9.8x10^-11^ | intergenic | 5.35 |
| **rs6677321** | 16149301 | 9151 | 0.967 | A/C | - | 0.785 | 9.8x10^-11^ | intergenic | 1.62 |
| **rs61782184** | 16150976 | 9146 | 0.964 | G/A | - | 0.785 | 8.6x10^-11^ | intergenic | 1.46 |
| **rs61782185** | 16150997 | 9146 | 0.964 | C/G | - | 0.785 | 8.6x10^-11^ | intergenic | 0.91 |
| **rs61782186** | 16151000 | 9146 | 0.964 | A/G | - | 0.785 | 8.6x10^-11^ | intergenic | 0.15 |
| **rs4661342** | 16160773 | 9365 | 0.976 | C/G | FLJ37453 | 0.755 | 9.5x10^-13^ | non_coding | 7.60 |
| **rs4661666** | 16162471 | 9146 | 0.967 | C/A | FLJ37453 | 0.785 | 9.5x10^-11^ | non_coding | 9.57 |
| **rs4661667** | 16162731 | 9133 | 0.966 | C/T | FLJ37453 | 0.784 | 7.4x10^-11^ | non_coding | 8.41 |
| **rs10927863** | 16165247 | 9146 | 0.967 | C/A | FLJ37453 | 0.785 | 9.5x10^-11^ | intron | 2.07 |
| **rs10927864** | 16165661 | 9146 | 0.967 | C/G | FLJ37453 | 0.785 | 9.5x10^-11^ | intron | 8.18 |
| **rs12135073** | 16167799 | 9406 | 0.978 | A/G | FLJ37453 | 0.759 | 1.8x10^-12^ | intron | 9.53 |
| **rs12744578** | 16173110 | 9360 | 0.977 | A/T | SPEN | 0.759 | 1.7x10^-12^ | upstream | 0.28 |
| **rs34224670** | 16175104 | 9072 | 0.962 | CT/C | SPEN | 0.780 | 3.4x10^-11^ | intron | 6.77 |
| **rs848217** | 16272250 | 8935 | 0.95 | A/G | ZBTB17 | 0.787 | 1.3x10^-11^ | synonymous | 0.50 |
| **rs4661674** | 16296039 | 9576 | 0.992 | A/G | ZBTB17 | 0.771 | 2.1x10^-12^ | intron | 0.19 |
| **rs71003222** | 16296982 | 9632 | 0.997 | G/GTTCA AGTGA | ZBTB17 | 0.770 | 1.6x10^-12^ | intron | 0.74 |
| **rs6684328** | 16297189 | 9505 | 0.99 | G/A | ZBTB17 | 0.772 | 4.1x10^-12^ | intron | 0.23 |
| **rs34763423** | 16297738 | 9524 | 0.98 | GA/G | ZBTB17 | 0.765 | 5.8x10^-13^ | intron | 4.68 |
| **rs10927875** | 16299312 | 9673 | 1 | C/T | ZBTB17 | 0.768 | 9.7x10^-13^ | intron | 5.50 |
| **rs698893** | 16302982 | 8955 | 0.94 | G/A | ZBTB17 | 0.783 | 9.6x10^-12^ | upstream | 7.88 |
| **rs34178196** | 16304398 | 9385 | 0.986 | G/A | ZBTB17 | 0.769 | 2.5x10^-12^ | upstream | 0.97 |
| **rs12138117** | 16305279 | 9496 | 0.987 | T/C | ZBTB17 | 0.773 | 5.1x10^-12^ | upstream | 0.29 |
| **rs3979179** | 16306846 | 9397 | 0.983 | G/A | ZBTB17 | 0.774 | 1.0x10^-11^ | upstream | 2.21 |
| **rs6656953** | 16307371 | 9522 | 0.989 | T/C | ZBTB17 | 0.771 | 3.4x10^-12^ | upstream | 0.77 |
| **rs848309** | 16308447 | 9139 | 0.963 | T/C | - | 0.781 | 4.2x10^-12^ | intergenic | 3.42 |
| **rs1739813** | 16311563 | 9095 | 0.961 | G/A | - | 0.781 | 4.7x10^-12^ | intergenic | 2.02 |
| **rs34764449** | 16312083 | 9381 | 0.982 | C/CA | - | 0.773 | 9.6x10^-12^ | intergenic | 10.1 |
| **rs3961710** | 16313172 | 9376 | 0.981 | C/T | - | 0.774 | 1.1x10^-11^ | intergenic | 9.56 |
| **rs4307563** | 16313788 | 9362 | 0.978 | C/T | - | 0.772 | 7.3x10^-12^ | intergenic | 2.46 |
| **rs10927878** | 16319957 | 8175 | 0.912 | C/T | - | 0.758 | 6.5x10^-12^ | intergenic | 3.80 |
| **rs34957618** | 16320499 | 8180 | 0.913 | G/C | - | 0.757 | 5.4x10^-12^ | intergenic | 3.91 |
| **rs1763619** | 16326935 | 8123 | 0.91 | A/G | C1orf64 | 0.753 | 1.9x10^-12^ | upstream | 0.68 |
| **rs1627145** | 16328116 | 8105 | 0.909 | C/T | C1orf64 | 0.752 | 1.5x10^-12^ | upstream | 2.26 |
| **rs1739833** | 16331108 | 8071 | 0.907 | T/C | C1orf64 | 0.751 | 1.6x10^-12^ | intron | 2.93 |
| **rs1739835** | 16335144 | 8045 | 0.905 | T/C | C1orf64 | 0.751 | 1.8x10^-12^ | downstream | 3.51 |
| **rs34033692** | 16335302 | 8057 | 0.905 | CA/C | C1orf64 | 0.751 | 1.7x10^-12^ | downstream | 1.03 |
| **rs1763610** | 16335527 | 8057 | 0.905 | G/C | HSPB7 | 0.751 | 1.7x10^-12^ | downstream | 0.73 |
| **rs1763607** | 16337509 | 8279 | 0.913 | G/A | HSPB7 | 0.767 | 3.2x10^-12^ | downstream | 7.33 |
| **rs1739837** | 16337933 | 8274 | 0.913 | C/T | HSPB7 | 0.767 | 3.1x10^-12^ | downstream | 2.50 |
| **rs1763606** | 16338282 | 8044 | 0.902 | A/G | HSPB7 | 0.733 | 6.2x10^-14^ | downstream | 0.35 |
| **rs6696912** | 16338474 | 8059 | 0.903 | T/C | HSPB7 | 0.733 | 5.8x10^-14^ | downstream | 0.66 |
| **rs1763605** | 16338925 | 8058 | 0.903 | T/G | HSPB7 | 0.734 | 6.6x10^-14^ | downstream | 5.46 |
| **rs1763603** | 16339809 | 7830 | 0.891 | T/C | HSPB7 | 0.736 | 4.3x10^-13^ | downstream | 7.86 |
| **rs60147323** | 16340048 | 8284 | 0.913 | A/AC | HSPB7 | 0.769 | 5.9x10^-12^ | downstream | 0.39 |
| **rs57972221** | 16340217 | 8041 | 0.899 | A/AACAGT GCTTGGC | HSPB7 | 0.778 | 8.4x10^-11^ | downstream | 0.79 |
| **rs1763601** | 16340460 | 8284 | 0.913 | G/T | HSPB7 | 0.769 | 6.0x10^-12^ | downstream | 6.68 |
| **rs1048334** | 16340761 | 8281 | 0.912 | T/C | HSPB7 | 0.768 | 4.5x10^-12^ | 3'_utr | 6.01 |
| **rs34035811** | 16340843 | 8055 | 0.903 | TC/T | HSPB7 | 0.734 | 7.8x10^-14^ | 3'_utr | 3.30 |
| **rs1048302** | 16340879 | 7913 | 0.895 | T/G | HSPB7 | 0.734 | 1.1x10^-13^ | 3'_utr | 4.85 |
| **rs1048261** | 16340951 | 8026 | 0.902 | A/T | HSPB7 | 0.734 | 7.1x10^-14^ | 3'_utr | 6.87 |
| **rs1048237** | 16341733 | 8273 | 0.912 | T/C | HSPB7 | 0.769 | 5.5x10^-12^ | 3'_utr | 9.91 |
| **rs1739840** | 16342237 | 8257 | 0.911 | A/G | HSPB7 | 0.769 | 6.4x10^-12^ | synonymous | 9 |
| **rs761759** | 16342350 | 8267 | 0.912 | A/T | HSPB7 | 0.770 | 7.1x10^-12^ | intron | 3.66 |
| **rs761760** | 16342419 | 8267 | 0.912 | A/G | HSPB7 | 0.770 | 7.1x10^-12^ | intron | 0.12 |
| **rs1763599** | 16342602 | 8258 | 0.911 | C/T | HSPB7 | 0.771 | 9.7x10^-12^ | intron | 7.67 |
| **rs1739841** | 16342727 | 8264 | 0.911 | A/G | HSPB7 | 0.770 | 8.2x10^-12^ | intron | 14.0 |
| **rs1739842** | 16342848 | 8264 | 0.911 | T/G | HSPB7 | 0.770 | 8.2x10^-12^ | intron | 8.06 |
| **rs1739843** | 16343254 | 8260 | 0.912 | T/C | HSPB7 | 0.768 | 4.7x10^-12^ | intron | 4.21 |
| **rs1763597** | 16343297 | 8258 | 0.912 | C/T | HSPB7 | 0.766 | 3.4x10^-12^ | intron | 8.63 |
| **rs1739844** | 16343322 | 8258 | 0.912 | T/C | HSPB7 | 0.766 | 3.4x10^-12^ | intron | 6.44 |
| **rs1763596** | 16343835 | 8171 | 0.909 | C/T | HSPB7 | 0.767 | 5.0x10^-12^ | intron | 5.09 |
| **rs732286** | 16344360 | 8228 | 0.911 | A/G | HSPB7 | 0.770 | 8.4x10^-12^ | synonymous | 21.7 |
| **rs945416** | 16344402 | 8227 | 0.911 | G/A | HSPB7 | 0.770 | 8.8x10^-12^ | synonymous | 16.7 |
| **rs1572381** | 16344494 | 8215 | 0.91 | G/A | HSPB7 | 0.770 | 8.2x10^-12^ | 5'_utr | 10.9 |
| **rs945417** | 16344625 | 7818 | 0.896 | C/G | HSPB7 | 0.773 | 3.5x10^-11^ | 5'_utr | 16.4 |
| **rs945418** | 16344730 | 7998 | 0.901 | T/C | HSPB7 | 0.736 | 1.6x10^-13^ | 5'_utr | 8.56 |
| **rs945419** | 16344858 | 7642 | 0.887 | C/T | HSPB7 | 0.775 | 8.0x10^-11^ | 5'_utr | 10.1 |
| **rs945420** | 16345208 | 7790 | 0.895 | G/A | HSPB7 | 0.775 | 5.2x10^-11^ | 5'_utr | 8.42 |
| **rs6660685** | 16346988 | 7857 | 0.894 | A/G | HSPB7 | 0.742 | 1.4x10^-12^ | upstream | 1.89 |
| **rs28579893** | 16347534 | 7820 | 0.892 | A/G | HSPB7 | 0.742 | 1.7x10^-12^ | upstream | 2.25 |
| **rs2009371** | 16348083 | 7826 | 0.892 | A/T | HSPB7 | 0.746 | 3.6x10^-12^ | upstream | 2.22 |
| **rs945425** | 16348412 | 7821 | 0.891 | T/C | HSPB7 | 0.744 | 2.9x10^-12^ | upstream | 5.70 |
| **rs9442214** | 16349460 | 7638 | 0.881 | T/C | HSPB7 | 0.746 | 7.7x10^-12^ | upstream | 3.74 |
| **rs2017583** | 16350028 | 7636 | 0.881 | A/G | HSPB7 | 0.745 | 7.2x10^-12^ | upstream | 5.40 |
| **rs9442216** | 16353400 | 7507 | 0.871 | T/C | CLCNKA | 0.738 | 3.3x10^-12^ | intron | 4.60 |
| **rs10927889** | 16354280 | 6816 | 0.849 | G/C | CLCNKA | 0.758 | 5.2x10^-11^ | intron | 1.89 |
| **rs2015509** | 16358148 | 7320 | 0.862 | T/C | CLCNKA | 0.740 | 8.2x10^-12^ | intron | 6.04 |
| **rs10803407** | 16358932 | 7247 | 0.859 | T/C | CLCNKA | 0.734 | 3.5x10^-12^ | splice_region | 4.79 |
| **rs9442217** | 16359123 | 7234 | 0.859 | A/G | CLCNKA | 0.734 | 3.8x10^-12^ | intron | 0.08 |
| **rs55731534** | 16362561 | 6692 | 0.828 | C/G | CLCNKA | 0.738 | 4.4x10^-11^ | downstream | 6.19 |

##### Table S3b. In Silico fine mapping at the *TTN* locus (P < 10^-6^)

|  | Position | N | Quality | Ref/Alt | Gene | OR | P-value | Consequence | CADD  score |
| --- | --- | --- | --- | --- | --- | --- | --- | --- | --- |
| **rs6735077** | 179381323 | 9401 | 0.97 | T/G | MIR548N | 0.804 | 2.5x10^-07^ | intron | 6.71 |
| **rs957875** | 179384417 | 9408 | 0.973 | T/C | TTN-AS1 | 0.803 | 1.9x10^-07^ | upstream | 1.98 |
| **rs3813250** | 179395958 | 9562 | 0.988 | T/C | TTN | 0.812 | 4.8x10^-07^ | synonymous | 4.20 |
| **rs3820978** | 179405807 | 9587 | 0.994 | A/G | TTN | 0.810 | 3.3x10^-07^ | intron | 0.58 |
| **rs3731750** | 179406003 | 9581 | 0.993 | C/A | TTN | 0.811 | 4.1x10^-07^ | splice_region | 16.0 |
| **rs9808377** | 179421694 | 9576 | 0.991 | A/G | TTN | 0.809 | 3.6x10^-07^ | missense | 0.58 |
| **rs2366751** | 179427186 | 9632 | 0.994 | A/G | TTN | 0.810 | 3.2x10^-07^ | synonymous | 13.2 |
| **rs3829746** | 179427536 | 9673 | 1 | T/C | TTN | 0.810 | 3.5x10^-07^ | missense | 13.8 |
| **rs3769864** | 179443217 | 9591 | 0.991 | T/G | TTN | 0.808 | 2.5x10^-07^ | intron | 7.69 |
| **rs4894029** | 179447848 | 9436 | 0.972 | T/C | TTN | 0.809 | 3.6x10^-07^ | synonymous | 15.8 |
| **rs16866400** | 179448911 | 9445 | 0.98 | G/A | TTN | 0.799 | 3.0x10^-07^ | intron | 1.04 |
| **rs2042996** | 179451420 | 9540 | 0.986 | G/A | TTN | 0.806 | 2.4x10^-07^ | missense | 20.1 |
| **rs1560221** | 179454394 | 9463 | 0.983 | A/G | TTN | 0.808 | 2.8x10^-07^ | synonymous | 10.1 |
| **rs2163009** | 179455207 | 9454 | 0.979 | T/C | TTN | 0.807 | 2.6x10^-07^ | synonymous | 8.17 |
| **rs6712785** | 179462142 | 9461 | 0.983 | A/G | TTN | 0.808 | 2.9x10^-07^ | intron | 1.94 |
| **rs12693162** | 179488194 | 9123 | 0.964 | C/T | TTN | 0.804 | 8.7x10^-07^ | intron | 18.7 |
| **rs12988307** | 179490478 | 9481 | 0.986 | T/C | TTN | 0.792 | 9.6x10^-08^ | intron | 0.90 |
| **rs36113194** | 179490785 | 9523 | 0.985 | C/T | TTN | 0.796 | 5.1x10^-08^ | intron | 4.58 |
| **rs16866420** | 179491235 | 9532 | 0.99 | T/G | TTN | 0.802 | 1.2x10^-07^ | intron | 7.23 |
| **rs2562845** | 179514433 | 9495 | 0.987 | T/C | TTN | 0.802 | 4.2x10^-07^ | intron | 6.04 |
| **rs71394376** | 179531078 | 9482 | 0.987 | A/AATGT | TTN | 0.812 | 7.0x10^-07^ | intron | 0.03 |
| **rs2472751** | 179539903 | 9527 | 0.988 | C/A | TTN | 0.798 | 7.7x10^-08^ | intron | 4.12 |
| **rs2255167** | 179558282 | 9574 | 0.993 | T/A | TTN | 0.801 | 3.6x10^-07^ | intron | 5.40 |
| **rs3045696** | 179562999 | 9615 | 0.995 | C/CTTAG | TTN | 0.813 | 6.3x10^-07^ | intron | 3.67 |

##### Table S3c. In Silico fine mapping at the *SLC39A8* locus (P < 10^-6^)

|  | Position | N | Quality | Ref/Alt | Gene | OR | P-value | Consequence | CADD score |
| --- | --- | --- | --- | --- | --- | --- | --- | --- | --- |
| **rs35225200** | 103146888 | 9673 | 0.912 | A/C | - | 1.35 | 6.1x10^-07^ | intergenic | 1.30 |
| **rs13107325** | 103188709 | 9673 | 1 | C/T | SLC39A8 | 1.35 | 6.1x10^-07^ | missense | 35 |
| **rs13135092** | 103198082 | 9673 | 0.982 | A/G | SLC39A8 | 1.35 | 6.1x10^-07^ | intronic | 9.74 |

##### Table S3d. In Silico fine mapping at the *MLIP* locus (P < 10^-7^)

|  | Position | N | Quality | Ref/Alt | Gene | OR | P-value | Consequence | CADD  score |
| --- | --- | --- | --- | --- | --- | --- | --- | --- | --- |
| **rs1830949** | 53964704 | 7527 | 0.882 | T/G | MLIP | 1.27 | 2.5x10^-09^ | intronic | 4.41 |
| **rs138844073** | 53966214 | 8593 | 0.917 | CTTAAT/C | MLIP | 1.23 | 5.2x10^-08^ | intronic | 11.4 |
| **rs1028804** | 53971401 | 7008 | 0.881 | G/A | MLIP | 1.26 | 8.9x10^-09^ | intronic | 4.06 |
| **rs1407229** | 53971921 | 6748 | 0.869 | T/C | MLIP | 1.29 | 5.3x10^-10^ | intronic | 0.51 |
| **rs10948798** | 53973770 | 8345 | 0.898 | C/T | MLIP | 1.25 | 9.3x10^-09^ | intronic | 1.16 |
| **rs35182047** | 53973844 | 6674 | 0.872 | C/CAT | MLIP | 1.30 | 2.5x10^-10^ | intronic | 2.33 |
| **rs670463** | 53977495 | 6782 | 0.879 | A/G | - | 1.26 | 1.4x10^-08^ | regulatory | 1.44 |
| **rs486135** | 53978817 | 8716 | 0.929 | T/G | - | 1.23 | 4.0x10^-08^ | regulatory | 1.66 |
| **rs28791933** | 53982630 | 7083 | 0.885 | G/A | MLIP | 1.26 | 6.8x10^-09^ | intronic | 2.26 |
| **rs4521576** | 53982990 | 8168 | 0.907 | A/C | MLIP | 1.25 | 4.1x10^-09^ | intronic | 1.28 |
| **rs1280949** | 53984899 | 7232 | 0.91 | C/T | MLIP | 1.25 | 1.2x10^-08^ | intronic | 4.22 |
| **rs1280948** | 53985075 | 7232 | 0.909 | A/C | MLIP | 1.25 | 1.2x10^-08^ | intronic | 0.31 |
| **rs1150887** | 53985153 | 7232 | 0.909 | C/T | MLIP | 1.25 | 1.2x10^-08^ | intronic | 2.25 |
| **rs1280947** | 53985167 | 7232 | 0.909 | A/G | MLIP | 1.25 | 1.2x10^-08^ | intronic | 1.97 |
| **rs1280946** | 53985339 | 7232 | 0.908 | T/C | MLIP | 1.25 | 1.2x10^-08^ | intronic | 1.99 |
| **rs4712056** | 53989526 | 9673 | 1 | G/A | MLIP | 1.19 | 4.6x10^-07^ | non_synonymous | 0.00 |
| **rs816364** | 53990465 | 8704 | 0.94 | G/A | - | 1.22 | 4.7x10^-08^ | regulatory | 8.48 |
| **rs816365** | 53991896 | 8702 | 0.939 | G/T | MLIP | 1.22 | 4.6x10^-08^ | intronic | 1.55 |
| **rs60281395** | 53992025 | 7313 | 0.915 | GA/G | MLIP | 1.25 | 1.4x10^-08^ | intronic | 0.37 |
| **rs816367** | 53995542 | 6938 | 0.878 | G/C | MLIP | 1.24 | 4.6x10^-08^ | intronic | 8.26 |
| **rs816378** | 53998123 | 6479 | 0.849 | T/G | MLIP | 1.29 | 1.4x10^-09^ | intronic | 1.78 |
| **rs816379** | 53999025 | 6990 | 0.875 | A/C | MLIP | 1.26 | 6.5x10^-09^ | intronic | 0.22 |
| **rs816373** | 54005043 | 6226 | 0.84 | A/G | MLIP | 1.25 | 8.8x10^-08^ | intronic | 4.46 |
| **rs816375** | 54007284 | 6228 | 0.84 | G/A | MLIP | 1.25 | 8.2x10^-08^ | intronic | 6.32 |
| **rs1407231** | 54009376 | 6218 | 0.839 | T/C | MLIP | 1.25 | 8.3x10^-08^ | intronic | 0.22 |
| **rs2797425** | 54012352 | 6220 | 0.835 | G/C | MLIP | 1.25 | 8.0x10^-08^ | intronic | 1.43 |
| **rs2145755** | 54013765 | 6208 | 0.834 | A/G | MLIP | 1.25 | 6.9x10^-08^ | intronic | 7.88 |
| **rs1407232** | 54016877 | 6199 | 0.832 | A/G | MLIP | 1.25 | 7.5x10^-08^ | intronic | 3.32 |
| **rs5876362** | 54017665 | 6256 | 0.842 | A/AGAAT | MLIP | 1.25 | 8.3x10^-08^ | intronic | 0.07 |
| **rs9395908** | 54019101 | 6239 | 0.84 | A/C | MLIP | 1.25 | 8.9x10^-08^ | intronic | 0.14 |
| **rs1407235** | 54022371 | 6213 | 0.835 | A/G | MLIP | 1.25 | 9.5x10^-08^ | intronic | 4.13 |
| **rs4301306** | 54024643 | 6248 | 0.841 | G/A | MLIP | 1.25 | 9.1x10^-08^ | intronic | 1.78 |
| **rs864642** | 54025859 | 6194 | 0.831 | T/A | MLIP | 1.25 | 8.2x10^-08^ | intronic | 7.88 |
| **rs2145759** | 54029152 | 6225 | 0.837 | G/C | MLIP | 1.25 | 6.8x10^-08^ | intronic | 0.39 |
| **rs816387** | 54039127 | 5684 | 0.806 | T/C | MLIP | 1.27 | 3.7x10^-08^ | intronic | 4.56 |
| **rs9296739** | 54054837 | 5678 | 0.804 | A/G | MLIP | 1.27 | 3.2x10^-08^ | intronic | 7.02 |

##### Table S3e. In Silico fine mapping at the *FLNC* locus (P < 10^-7^)

|  | Position | N | Quality | Ref/Alt | Gene | OR | P-value | Consequence | CADD  score |
| --- | --- | --- | --- | --- | --- | --- | --- | --- | --- |
| **exm656431** | 128488734 | 9673 | 1 | G/A | FLNC | 0.652 | 9.0x10^-11^ | non_synonymous | 11.4 |
| **rs72348656** | 128498630 | 8723 | 0.765 | TACACAC/T | FLNC | 0.663 | 1.4x10^-09^ | 3' utr | 15.4 |
| **rs3800585** | 128498791 | 8657 | 0.766 | C/T | FLNC | 0.654 | 3.3x10^-10^ | 3' utr | 6.60 |

##### Table S3f. In Silico fine mapping at the *BAG3* locus (P < 10^-7^)

|  | Position | N | Quality | Ref/Alt | Gene | OR | P-value | Consequence | CADD  score |
| --- | --- | --- | --- | --- | --- | --- | --- | --- | --- |
| **rs3847489** | 121417338 | 7956 | 0.885 | T/G | - | 1.256 | 5.4x10^-09^ | regulatory | 5.18 |
| **rs17099139** | 121419487 | 5292 | 0.716 | C/G | - | 0.627 | 1.2x10^-16^ | regulatory | 2.42 |
| **rs4752339** | 121421269 | 7539 | 0.87 | T/A | BAG3 | 1.247 | 3.4x10^-08^ | intronic | 12.1 |
| **rs7095308** | 121426312 | 5337 | 0.728 | G/A | BAG3 | 0.618 | 1.1x10^-17^ | intronic | 11.0 |
| **rs10886529** | 121427082 | 7643 | 0.874 | G/A | - | 1.275 | 8.9x10^-10^ | regulatory | 0.85 |
| **rs4752340** | 121427877 | 6947 | 0.861 | C/T | BAG3 | 1.288 | 8.5x10^-10^ | intronic | 4.62 |
| **rs2234962** | 121429633 | 9673 | 1 | T/C | BAG3 | 0.620 | 1.7x10^-25^ | missense | 24.2 |
| **rs2289307** | 121582556 | 9537 | 0.985 | T/C | INPP5F | 1.228 | 6.1x10^-09^ | intronic | 5.83 |
| **rs4752345** | 121584676 | 9528 | 0.975 | T/C | MCMBP | 1.232 | 4.1x10^-09^ | downstream | 12.6 |
| **rs3188055** | 121586882 | 9673 | 1 | A/G | INPP5F | 1.223 | 1.1x10^-08^ | missense | 0.07 |
| **rs2244165** | 121597160 | 8879 | 0.94 | A/G | MCMBP | 1.244 | 7.7x10^-09^ | intronic | 1.37 |
| **rs2247724** | 121605191 | 8533 | 0.932 | G/C | MCMBP | 1.245 | 1.8x10^-08^ | intronic | 3.58 |
| **rs2456713** | 121605378 | 8904 | 0.935 | A/G | MCMBP | 1.241 | 9.0x10^-09^ | intronic | 1.75 |
| **rs2456728** | 121623844 | 8849 | 0.934 | A/G | - | 1.236 | 2.5x10^-08^ | regulatory | 1.7 |
| **rs2456727** | 121624298 | 8489 | 0.928 | T/C | MCMBP | 1.240 | 3.8x10^-08^ | intronic | 3.46 |
| **rs2984243** | 121626737 | 8464 | 0.927 | A/G | MCMBP | 1.236 | 7.8x10^-08^ | intronic | 0.00 |
| **rs2456717** | 121638633 | 8827 | 0.935 | A/G | MCMBP | 1.233 | 3.9x10^-08^ | intronic | 0.58 |
| **rs2939932** | 121640211 | 8454 | 0.924 | T/C | MCMBP | 1.235 | 8.8x10^-08^ | intronic | 4.08 |
| **rs2475317** | 121642837 | 8850 | 0.935 | T/C | MCMBP | 1.231 | 4.7x10^-08^ | intronic | 4.38 |
| **rs2475313** | 121646618 | 8846 | 0.935 | C/G | MCMBP | 1.231 | 4.4x10^-08^ | intronic | 0.57 |
| **rs2475312** | 121647627 | 8454 | 0.924 | A/T | SEC23IP | 1.235 | 8.8x10^-08^ | upstream | 0.81 |
| **rs2475311** | 121647728 | 8454 | 0.924 | C/T | SEC23IP | 1.235 | 8.8x10^-08^ | upstream | 1.54 |
| **rs2475309** | 121649505 | 8850 | 0.935 | T/C | SEC23IP | 1.231 | 4.7x10^-08^ | upstream | 0.45 |

#####

##### Table S3g. In Silico fine mapping at the *ALPK3* locus (P < 10^-7^)

|  | Position | N | Quality | Ref/Alt | Gene | OR | P-value | Consequence | CADD  score |
| --- | --- | --- | --- | --- | --- | --- | --- | --- | --- |
| **rs4536430** | 84917314 | 8114 | 0.903 | T/C | GOLGA6L4 | 1.33 | 4.6x10^-08^ | downstream | 3.07 |
| **rs2401448** | 84918172 | 8639 | 0.943 | G/T | GOLGA6L4 | 1.31 | 8.1x10^-08^ | downstream | 0.03 |
| **rs62028133** | 84919375 | 8589 | 0.942 | A/C | - | 1.31 | 9.5x10^-08^ | intergenic | 0.02 |
| **rs17589320** | 84919996 | 8641 | 0.944 | C/A | - | 1.31 | 8.5x10^-08^ | intergenic | 1.00 |
| **rs35203246** | 84920758 | 8639 | 0.944 | G/A | - | 1.31 | 9.5x10^-08^ | intergenic | 0.02 |
| **rs4502182** | 84923520 | 8621 | 0.943 | A/G | - | 1.31 | 7.3x10^-08^ | intergenic | 0.11 |
| **rs4842846** | 84923998 | 8636 | 0.944 | G/A | - | 1.31 | 7.8x10^-08^ | intergenic | 9.14 |
| **rs35474552** | 84927981 | 8622 | 0.941 | C/T | - | 1.31 | 7.7x10^-08^ | intergenic | 2.85 |
| **rs4499200** | 84928263 | 8622 | 0.941 | T/G | - | 1.31 | 7.7x10^-08^ | intergenic | 3.63 |
| **rs35156560** | 84929063 | 8579 | 0.935 | A/T | - | 1.31 | 9.1x10^-08^ | intergenic | 4.68 |
| **rs149987188** | 84936529 | 8525 | 0.936 | G/A | - | 1.31 | 9.1x10^-08^ | intergenic | 1.74 |
| **rs199758543** | 84936887 | 8529 | 0.937 | C/G | - | 1.31 | 7.9x10^-08^ | intergenic | 4.58 |
| **rs35957708** | 84939711 | 8512 | 0.936 | A/G | - | 1.31 | 8.6x10^-08^ | intergenic | 0.38 |
| **rs2896002** | 84945121 | 8539 | 0.936 | A/G | - | 1.31 | 8.0x10^-08^ | intergenic | 1.83 |
| **rs11632668** | 84948687 | 8525 | 0.936 | T/C | - | 1.31 | 9.1x10^-08^ | intergenic | 2.72 |
| **rs35986397** | 84955107 | 8505 | 0.935 | G/A | - | 1.31 | 1.0x10^-07^ | intergenic | 1.59 |
| **rs62029597** | 84956479 | 8535 | 0.934 | G/A | - | 1.31 | 9.6x10^-08^ | intergenic | 0.75 |
| **rs2036949** | 85163605 | 9414 | 0.984 | T/C | ZSCAN2 | 1.29 | 8.9x10^-08^ | downstream | 0.56 |
| **rs17598114** | 85171317 | 9352 | 0.981 | C/T | ZSCAN2 | 1.30 | 6.8x10^-08^ | downstream | 1.87 |
| **rs17532346** | 85171495 | 9352 | 0.981 | A/C | ZSCAN2 | 1.30 | 6.8x10^-08^ | downstream | 5.77 |
| **rs143475480** | 85238906 | 9422 | 0.985 | C/CATTTTCT | SEC11A | 1.30 | 6.3x10^-08^ | intron | 9.08 |
| **rs62021226** | 85268036 | 9343 | 0.982 | T/C | - | 1.30 | 4.8x10^-08^ | intergenic | 1.95 |
| **rs12916867** | 85270275 | 9345 | 0.98 | T/C | - | 1.30 | 4.8x10^-08^ | intergenic | 5.56 |
| **rs34570071** | 85280212 | 9200 | 0.974 | T/A | - | 1.30 | 9.9x10^-08^ | intergenic | 0.28 |
| **rs60957376** | 85280792 | 9320 | 0.979 | T/G | - | 1.30 | 6.1x10^-08^ | intergenic | 4.47 |
| **rs11633788** | 85305142 | 8427 | 0.936 | T/A | ZNF592 | 1.32 | 8.7x10^-08^ | intron | 9.30 |
| **rs12908549** | 85322351 | 9318 | 0.979 | C/G | ZNF592 | 1.30 | 7.3x10^-08^ | intron | 2.82 |
| **rs35726233** | 85337800 | 9317 | 0.98 | C/T | ZNF592 | 1.30 | 8.3x10^-08^ | intron | 0.13 |
| **rs35960805** | 85343980 | 9326 | 0.98 | T/G | ZNF592 | 1.30 | 7.1x10^-08^ | intron | 5.94 |
| **rs12912388** | 85344550 | 9326 | 0.98 | T/A | ZNF592 | 1.30 | 7.1x10^-08^ | intron | 19.6 |
| **rs17601029** | 85347709 | 9013 | 0.961 | A/G | ZNF592 | 1.32 | 2.4x10^-08^ | 3'_utr | 1.63 |
| **rs3803403** | 85383145 | 9673 | 1 | C/G | ALPK3 | 1.28 | 2.8x10^-07^ | missense | 3.00 |

##### Table S3h. In Silico fine mapping at the *FHOD3* locus (P < 10^-7^)

|  | Position | N | Quality | Ref/Alt | Gene | OR | P-value | Consequence | CADD score |
| --- | --- | --- | --- | --- | --- | --- | --- | --- | --- |
| **rs34100890** | 34261581 | 4665 | 0.739 | AG/A | FHOD3 | 0.775 | 9.6x10^-07^ | intronic | 1.63 |
| **rs671447** | 34262350 | 4688 | 0.741 | C/T | FHOD3 | 0.775 | 9.2x10^-07^ | intronic | 3.60 |
| **rs627756** | 34265279 | 4931 | 0.747 | G/A | FHOD3 | 0.774 | 5.5x10^-07^ | intronic | 2.67 |
| **rs500559** | 34270194 | 5925 | 0.776 | A/G | FHOD3 | 0.777 | 1.4x10^-07^ | intronic | 1.06 |
| **rs4603639** | 34292319 | 6159 | 0.793 | A/G | FHOD3 | 0.798 | 8.3x10^-07^ | intronic | 2.31 |
| **rs4477795** | 34292366 | 6275 | 0.79 | C/T | FHOD3 | 0.794 | 4.9x10^-07^ | intronic | 1.20 |
| **rs72232902** | 34293994 | 6694 | 0.808 | GTC/G | FHOD3 | 0.794 | 4.2x10^-07^ | intronic | 1.09 |
| **rs12605417** | 34294896 | 6510 | 0.8 | C/T | FHOD3 | 0.798 | 8.8x10^-07^ | intronic | 4.64 |
| **rs7240383** | 34298732 | 8650 | 0.91 | G/C | - | 0.812 | 6.8x10^-08^ | regulatory | 4.68 |
| **rs35045610** | 34299383 | 8637 | 0.913 | CT/C | FHOD3 | 0.814 | 8.5x10^-08^ | intronic | 9.82 |
| **rs11081962** | 34299757 | 7753 | 0.865 | G/A | FHOD3 | 0.811 | 8.1x10^-07^ | intronic | 1.35 |
| **rs9956820** | 34301780 | 8646 | 0.909 | A/T | FHOD3 | 0.810 | 4.5x10^-08^ | intronic | 3.28 |
| **rs9962453** | 34309966 | 8146 | 0.897 | C/T | FHOD3 | 0.817 | 2.7x10^-07^ | intronic | 5.24 |
| **rs3744903** | 34310668 | 7835 | 0.875 | C/T | FHOD3 | 0.811 | 7.3x10^-07^ | synonymous | 18.2 |
| **rs4799884** | 34313301 | 8904 | 0.942 | G/A | FHOD3 | 0.818 | 1.4x10^-07^ | intronic | 0.31 |
| **rs12604388** | 34315711 | 8945 | 0.947 | C/T | FHOD3 | 0.820 | 1.7x10^-07^ | intronic | 4.90 |
| **rs4799887** | 34319157 | 9362 | 0.961 | C/T | FHOD3 | 0.826 | 3.2x10^-07^ | intronic | 0.99 |
| **rs11660290** | 34321757 | 8863 | 0.934 | G/T | FHOD3 | 0.820 | 2.0x10^-07^ | intronic | 0.51 |
| **rs2303510** | 34324091 | 9673 | 1 | G/A | FHOD3 | 0.824 | 1.4x10^-07^ | missense | 22.8 |
