# Supplementary material for: Exome-wide association study reveals novel susceptibility genes to sporadic dilated cardiomyopathy
Source: PLoS One. 2017 Mar 15;12(3):e0172995. doi: 10.1371/journal.pone.0172995 (PMC5351854; doi:10.1371/journal.pone.0172995)
Supplement: S4 Table — (DOCX) [file pone.0172995.s008.docx]

##### Table S4. *SKAT* analysis of variants in genes known to be associated with familial cardiomyopathy

| genes | number of variant (rare f<0.01) | All variants P-value | Rare variants P-value | Common variants P-value |
| --- | --- | --- | --- | --- |
| ABCC9 | 9 (8) | 0.133 | 0.072 | 0.36 |
| ACTN2 | 12 (11) | 0.7716 | 0.62 | 0.71 |
| ANKRD1 | 7 (7) | 0.5783 | 0.58 | NA |
| CASQ2 | 8 (6) | 0.06264 | 0.65 | 0.022 |
| CAV3 | 4 (4) | 0.003762 | 0.0038 | NA |
| COX15 | 6 (4) | 0.4401 | 0.49 | 0.31 |
| CRYAB | 2 (2) | 0.1636 | 0.16 | NA |
| CSRP3 | 3 (3) | 0.2788 | 0.28 | NA |
| DES | 4 (3) | 0.1579 | 0.078 | 0.43 |
| DMD | 31 (22) | 0.6185 | 0.56 | 0.53 |
| DSC2 | 14 (11) | 0.03711 | 0.034 | 0.19 |
| DSG2 | 18 (14) | 0.01831 | 0.14 | 0.02 |
| DSP | 26 (21) | 0.4717 | 0.29 | 0.63 |
| DTNA | 7 (6) | 0.04183 | 0.023 | 0.27 |
| EMD | 1 (1) | 0.6004 | 0.6 | NA |
| EYA4 | 5 (3) | 0.2221 | 0.47 | 0.13 |
| FHL2 | 4 (3) | 0.9233 | 0.75 | 0.89 |
| FKTN | 8 (4) | 0.02583 | 0.091 | 0.039 |
| GLA | 4 (4) | 0.3688 | 0.37 | NA |
| JUP | 6 (5) | 0.02628 | 0.064 | 0.05 |
| LAMA4 | 25 (20) | 0.5391 | 0.75 | 0.28 |
| LAMP2 | 5 (4) | 0.1692 | 0.062 | 0.68 |
| LDB3 | 13 (13) | 0.4265 | 0.43 | NA |
| LMNA | 3 (2) | 1 | 1 | 0.82 |
| MYBPC3 | 21 (17) | 0.000746 | 0.67 | 5.7x10^-5^ |
| MYH6 | 15 (13) | 0.03837 | 0.029 | 0.19 |
| MYH7 | 3 (1) | 0.4211 | 0.34 | 0.38 |
| MYL3 | 0 (0) | 1 | 1 | 1 |
| MYLK2 | 4 (4) | 0.8404 | 0.84 | NA |
| MYOZ2 | 0 (0) | 1 | 1 | 1 |
| NEXN | 4 (4) | 0.9862 | 0.99 | NA |
| PKP2 | 11 (9) | 0.6642 | 0.72 | 0.41 |
| PRKAG2 | 7 (4) | 0.2481 | 0.09 | 0.75 |
| PSEN1 | 2 (1) | 0.09812 | 0.96 | 0.031 |
| PSEN2 | 2 (2) | 0.04397 | 0.044 | NA |
| RBM20 | 11 (11) | 0.00428 | 0.0043 | NA |
| RYR2 | 19 (11) | 0.2789 | 0.19 | 0.46 |
| SCN5A | 20 (13) | 0.8298 | 0.89 | 0.52 |
| SDHA | 2 (1) | 0.2163 | 0.87 | 0.081 |
| SGCD | 5 (2) | 0.6638 | 1 | 0.36 |
| SYNE1 | 112 (88) | 0.9843 | 0.96 | 0.89 |
| SYNE2 | 92 (74) | 0.5604 | 0.23 | 0.87 |
| TCAP | 2 (2) | 0.3052 | 0.31 | NA |
| TMEM43 | 9 (5) | 0.2103 | 0.054 | 0.96 |
| TMPO | 15 (13) | 0.06557 | 0.041 | 0.29 |
| TNNI3 | 3 (1) | 0.3661 | 0.27 | 0.4 |
| TNNT2 | 4 (3) | 0.5735 | 0.33 | 0.99 |
| TPM1 | 2 (1) | 0.412 | 0.3 | 0.4 |
| TTR | 6 (5) | 0.9254 | 0.77 | 0.94 |
| VCL | 12 (12) | 0.0625 | 0.062 | NA |

All variants available in the data set present on 50 genes that have been reported to be associated with familial cardiomyopathy (see text) were selected and analyzed with *SKAT*. Genes identified in the present GWAS were excluded from this analysis.
