# Supplementary material for: Exome-wide association study reveals novel susceptibility genes to sporadic dilated cardiomyopathy
Source: PLoS One. 2017 Mar 15;12(3):e0172995. doi: 10.1371/journal.pone.0172995 (PMC5351854; doi:10.1371/journal.pone.0172995)
Supplement: S7 Table — (DOCX) [file pone.0172995.s011.docx]

## S7 Table. Association statistics comparison with and without the 85 FDCM cases

|  | **All cases** | | **Without 85 FDCM cases** | |  | |
| --- | --- | --- | --- | --- | --- | --- |
| SNP rs number | MAF | P-value | MAF | P-value | Gene |  |
| rs848210 | A: 0.44 | 6.3x10^-07^ | A: 0.44 | 1.8x10^-06^ | SPEN |  |
| rs10927875 | A: 0.32 | 8.1x10^-13^ | A: 0.31 | 5.2x10^-12^ | ZBTB17 |  |
| rs3829746 | G: 0.24 | 3.4x10^-07^ | G: 0.23 | 5.8x10^-07^ | TTN |  |
| rs13107325 | A: 0.08 | 6.0x10^-07^ | A: 0.08 | 3.3x10^-06^ | SLC39A8 |  |
| rs4712056 | G: 0.35 | 5.1x10^-07^ | G: 0.35 | 6.3x10^-07^ | MLIP |  |
| rs2291569 | A: 0.08 | 8.7x10^-11^ | A: 0.08 | 6.5x10^-11^ | FLNC |  |
| rs2234962 | G: 0.19 | 1.7x10^-25^ | G: 0.19 | 7.6x10^-25^ | BAG3 |  |
| rs3188055 | G: 0.33 | 1.1x10^-08^ | G: 0.34 | 4.5x10^-08^ | INPP5F |  |
| *rs1051168 | A: 0.29 | 4.1x10^-07^ | A: 0.30 | 2.7x10^-07^ | NMB |  |
| *rs3803403 | G: 0.29 | 2.9x10^-07^ | G: 0.30 | 2.0x10^-07^ | ALPK3 |  |
| rs2303510 | A: 0.32 | 1.5x10^-07^ | A: 0.31 | 3.2x10^-07^ | FHOD3 |  |

MAF, minor allele frequency. For gene symbols, refer to table 1. *For rs1051168 (*NMB*) and rs3803403 (*ALPK3*) the result of the dominant model is shown as it is better supported than the additive test which is presented for all other variants.
