# Supplementary material for: Correction: Exome-wide association study reveals novel susceptibility genes to sporadic dilated cardiomyopathy
Source: PLoS One. 2020 Feb 14;15(2):e0229472. doi: 10.1371/journal.pone.0229472 (PMC7021299; doi:10.1371/journal.pone.0229472)
Supplement: S1 File — (PPTX) [file pone.0229472.s001.pptx]

## Slide 1
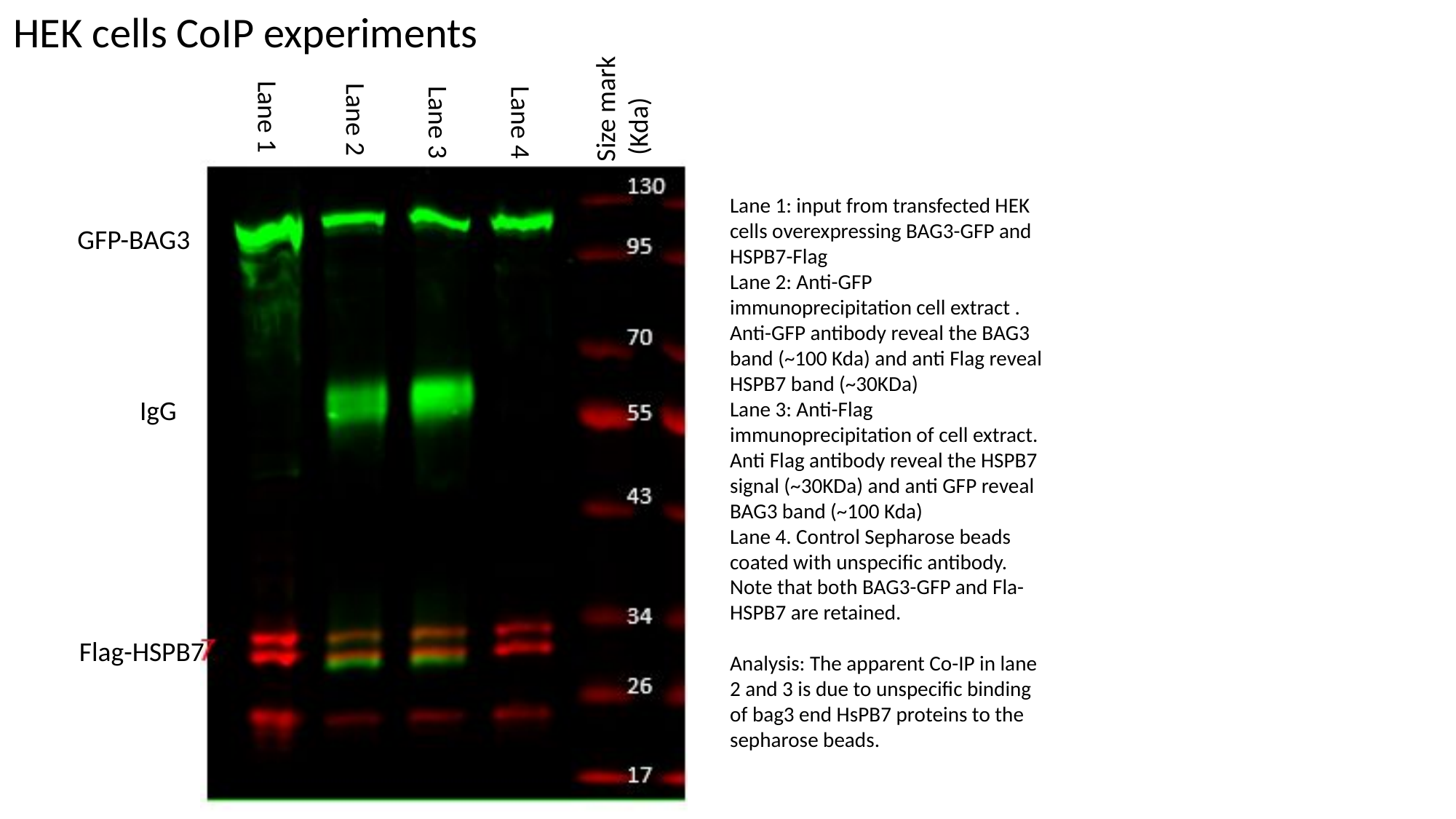

HEK cells CoIP experiments
Size mark
 (Kda)
Lane 1
Lane 2
Lane 3
Lane 4
Lane 1: input from transfected HEK cells overexpressing BAG3-GFP and HSPB7-Flag
Lane 2: Anti-GFP immunoprecipitation cell extract . Anti-GFP antibody reveal the BAG3 band (~100 Kda) and anti Flag reveal HSPB7 band (~30KDa)
Lane 3: Anti-Flag immunoprecipitation of cell extract. Anti Flag antibody reveal the HSPB7 signal (~30KDa) and anti GFP reveal BAG3 band (~100 Kda)
Lane 4. Control Sepharose beads coated with unspecific antibody. Note that both BAG3-GFP and Fla-HSPB7 are retained.
Analysis: The apparent Co-IP in lane 2 and 3 is due to unspecific binding of bag3 end HsPB7 proteins to the sepharose beads.
GFP-BAG3
IgG
Flag-HSPB7
