# Supplementary material for: Correction: Exome-wide association study reveals novel susceptibility genes to sporadic dilated cardiomyopathy
Source: PLoS One. 2020 Feb 14;15(2):e0229472. doi: 10.1371/journal.pone.0229472 (PMC7021299; doi:10.1371/journal.pone.0229472)
Supplement: S2 File — (PPTX) [file pone.0229472.s002.pptx]

## Slide 1
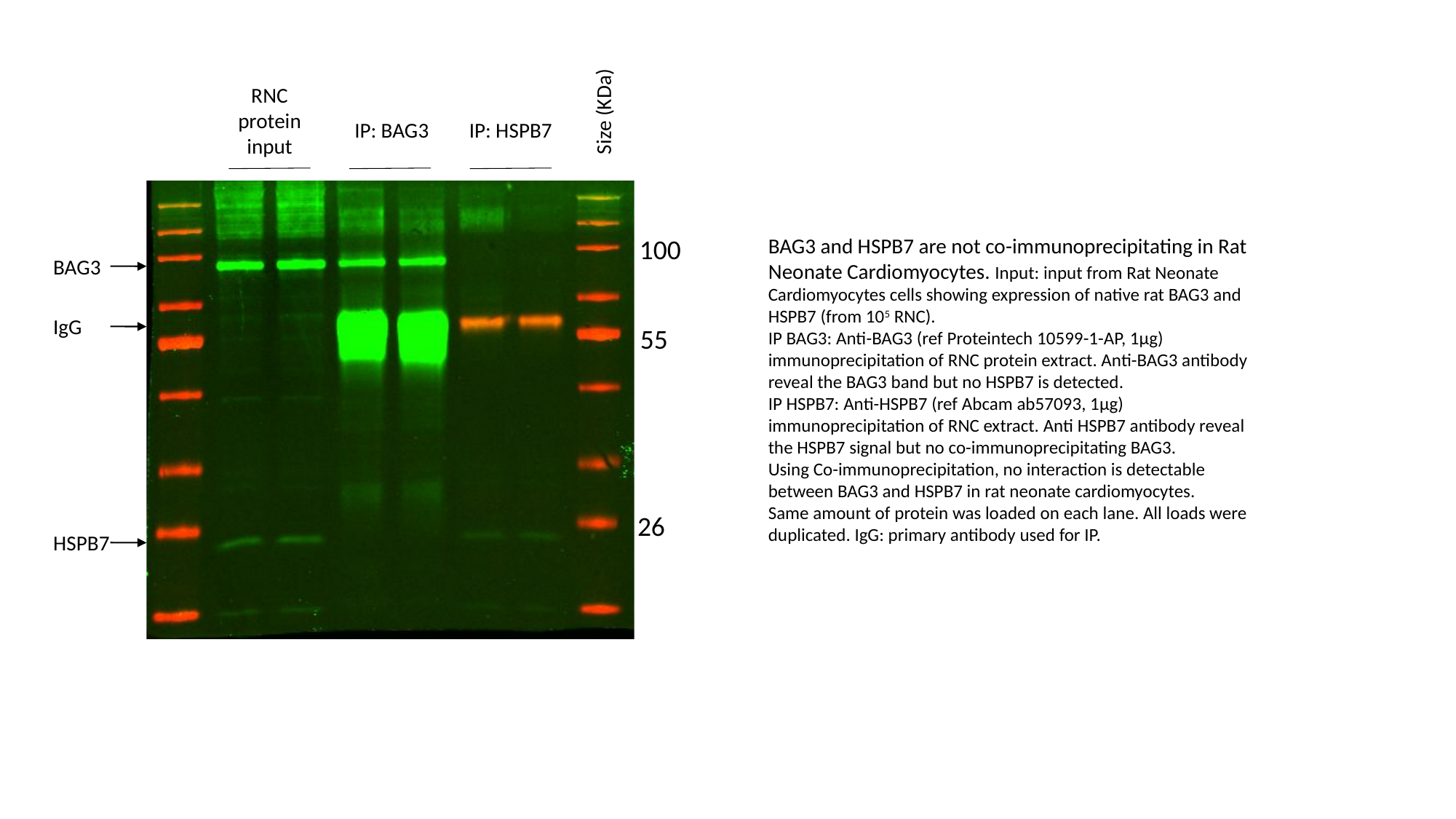

RNC protein input
Size (KDa)
IP: BAG3
IP: HSPB7
100
BAG3 and HSPB7 are not co-immunoprecipitating in Rat Neonate Cardiomyocytes. Input: input from Rat Neonate Cardiomyocytes cells showing expression of native rat BAG3 and HSPB7 (from 105 RNC).
IP BAG3: Anti-BAG3 (ref Proteintech 10599-1-AP, 1µg) immunoprecipitation of RNC protein extract. Anti-BAG3 antibody reveal the BAG3 band but no HSPB7 is detected.
IP HSPB7: Anti-HSPB7 (ref Abcam ab57093, 1µg) immunoprecipitation of RNC extract. Anti HSPB7 antibody reveal the HSPB7 signal but no co-immunoprecipitating BAG3.
Using Co-immunoprecipitation, no interaction is detectable between BAG3 and HSPB7 in rat neonate cardiomyocytes.
Same amount of protein was loaded on each lane. All loads were duplicated. IgG: primary antibody used for IP.
BAG3
IgG
55
26
HSPB7
